# Role of Group 12 Metals in the Reduction of H_2_O_2_ by Santi’s Reagent: A Computational Mechanistic Investigation

**DOI:** 10.1021/acs.inorgchem.3c02568

**Published:** 2023-09-28

**Authors:** Davide Zeppilli, Andrea Madabeni, Luca Sancineto, Luana Bagnoli, Claudio Santi, Laura Orian

**Affiliations:** † Dipartimento di Scienze Chimiche, Università degli Studi di Padova, Via Marzolo 1, 35131 Padova, Italy; ‡ Gruppo di Catalisi Sintesi e Chimica Organica Verde Dipartimento di Scienze Farmaceutiche, Università degli Studi di Perugia, Via del Liceo 1, 06122 Perugia, Italy

## Abstract

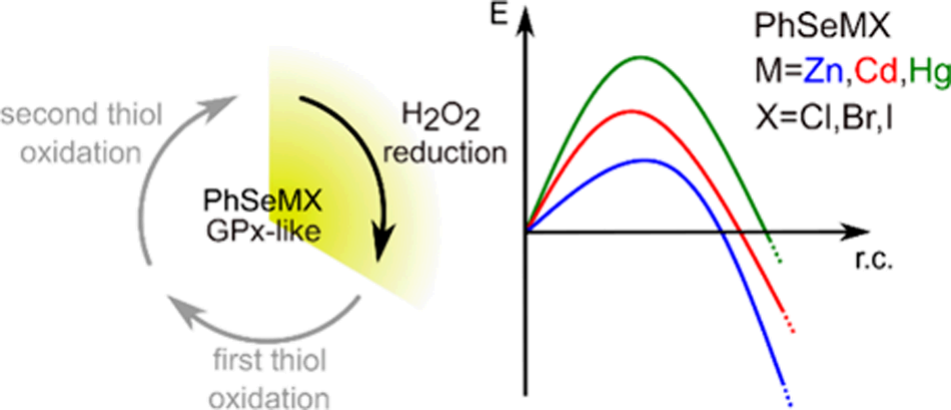

PhSeZnCl, which is also known as Santi’s reagent, can catalyze the reduction of hydrogen peroxide by thiols with a GPx-like mechanism. In this work, the first step of this catalytic cycle, i.e., the reduction of H_2_O_2_ by PhSeZnCl, is investigated *in silico* using state-of-the-art density functional theory calculations. Then, the role of the metal is evaluated by replacing Zn with its group 12 siblings (Cd and Hg). The thermodynamic and kinetic factors favoring Zn are elucidated. Furthermore, the role of the halogen is considered by replacing Cl with Br in all three metal compounds, and this turns out to be negligible. Finally, the overall GPx-like mechanism of PhSeZnCl and PhSeZnBr is discussed by evaluating the energetics of the mechanistic path leading to the disulfide product.

## Abstract 2

PhSeMX (M = Zn, Cd, Hg; X= Cl, Br, I) can reduce hydrogen peroxide in a three-step GPx-like mechanism. The first one, i.e., the proper reduction of the peroxide, is affected by the choice of the group 12 metal. In all cases, the reaction is energetically favored, but Zn is associated with the lowest activation energy, whereas the highest one is observed in the presence of Hg. In contrast, the choice of the halogen does not affect the process.

## 1 Introduction

**Scheme 1:**
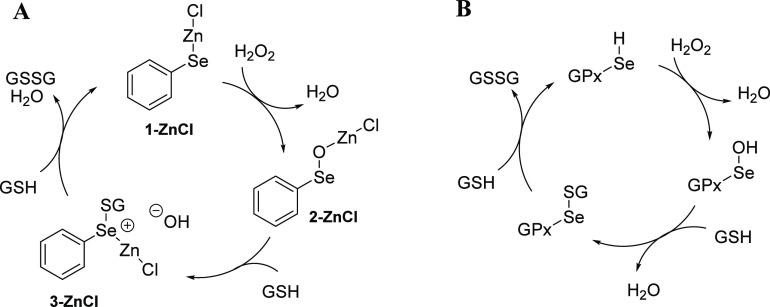
(A) GPx-like Mechanism of PhSeZnCl according to the Work of Santi *et al.*;^38^ (B) Catalytic Cycle of Glutathione Peroxidase (GPx) according to the Description of Flohé *et al*.^40^

PhSeZnCl (**1-ZnCl**, Scheme 1A) is a zinc selenolate prepared via the oxidative insertion of elemental zinc into the selenium-chloride bond of the commercially available PhSeCl in refluxing THF.^1^ It was the first reported bench-stable nucleophilic reagent largely used in several “on water” reactions and other efficient nucleophilic selenenylations.^2−4^

A similar zinc selenolate was first proposed as an intermediate nucleophilic reagent for the synthesis of allyl halides^5^ and α-selenocarbonyl derivatives.^6^ Then, with a novel protocol,^1^**1-ZnCl** was isolated as a bench-stable solid; furthermore, PhSeZnBr (**1-ZnBr**) was also synthesized with a similar protocol, starting from PhSeBr. These compounds have been widely used as nucleophilic selenenylating agents in organoselenium chemistry, like in vinylic substitutions^7^ and in ring openings of epoxides and aziridines.^8^ Other applications of these reagents include the synthesis of selenosteroids,^9,10^ the functionalization of polymers,^11^ and the synthesis of a fluorescent probe to detect glutathione.^12^ Finally, **1-ZnCl** has become commercially available in 2016, when it was named, for the first time, Santi’s reagent.^13^

An important aspect of the organoselenium chemistry is its unexpected applicability in green protocols;^14^ selenocompounds can be used as catalysts or electrophilic, nucleophilic, or radical reagents to perform direct functionalization of organic substrates by circumventing the need for protection–deprotection steps, which can be critical when aiming to create environmentally friendly chemical procedures.^15−19^ Particularly, the green features of organoselenides’ reactivity are the possibility to use safe, nontoxic, and nonconventional media, like water,^20−22^ ionic liquids,^23,24^ glycerol,^25,26^ or solvent-free conditions;^27,28^ other advantages are the use of alternative sources of energy, like microwaves,^29,30^ ultrasounds,^31−33^ or electrochemistry;^34^ and the application in bioinspired catalysis.^35−37^

As nucleophilic selenenylating reagent, **1-ZnCl** was also used as catalyst in the oxidation of thiols to disulfides in the presence of air or peroxides.^38^ This catalytic activity is efficient with many thiols, including glutathione (GSH); notably, GSH is not oxidized when exposed to air unless **1-ZnCl** is added; finally, Santi’s reagent also accelerates the oxidation of GSH by hydrogen peroxide (H_2_O_2_).^38^ This last reaction mimics the mechanism of the selenoprotein glutathione peroxidases (GPx, Scheme 1B).^39−42^ These enzymes catalyze the reduction of hydroperoxides using GSH as cofactor; in particular, in the oxidative step, the selenocysteine present in the active site is oxidized to selenenic acid by the hydroperoxide. Then, in the two-step reductive stage, 2 equiv of GSH restore the native form of the enzyme, passing through a selenosulfide intermediate.^43^ The proposed mechanism for the GPx-like mechanism of **1-ZnCl**(38) is shown in Scheme 1A, and the Zn^2+^ Lewis acid plays a significant role. Also in this case, the first step of the catalytic cycle is the reduction of H_2_O_2_ by **1-ZnCl** leading to water and to a peculiar product, i.e., **2-ZnCl.** In analogy with the direct oxidation of organochalcogen substrates to the corresponding oxides,^44^ we expected that the oxidation of a chalcogen containing ligand would lead, in this case, to the zinc selenenate-κSe. In contrast, the mechanism proposed by Santi et al.^38^ starts with the insertion of oxygen into the Zn–Se bond, forming an oxygen bridge between the metal and the chalcogen and thus resulting in the zinc selenenate-κO. The following steps show close analogies to those of GPx (Scheme 1B). First, GSH binds to the Se atom with the formation of the Se–S bond characterizing selenosulfide **3-ZnCl**. Then, a second GSH attacks the sulfur center, leading to the cleavage of the oxidized glutathione (GSSG) and to the regeneration of the catalyst **1-ZnCl**.

**Scheme 2:**
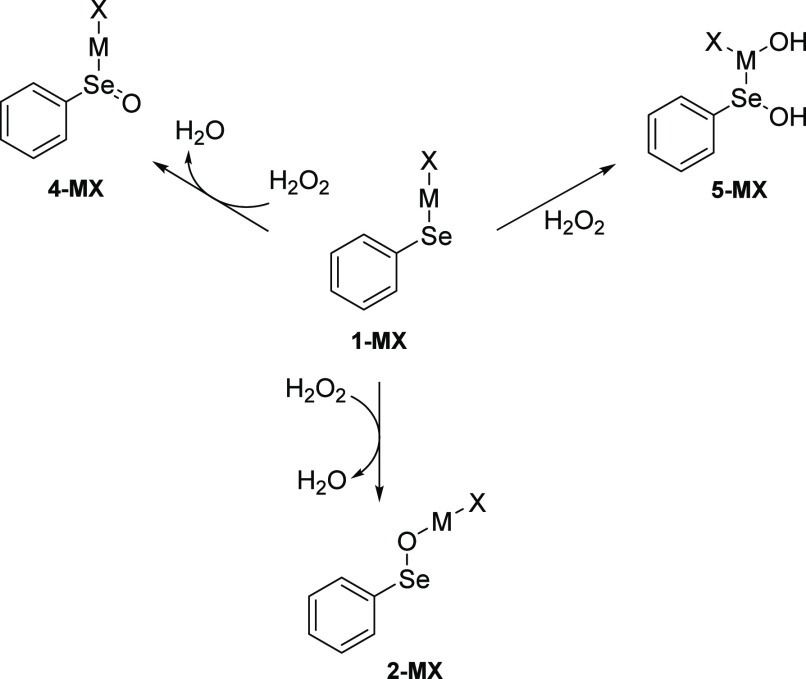
Different Scenarios for the Reduction of H_2_O_2_ by PhSeMX **1-MX** (M = Zn, Cd, Hg; X= Cl, Br, I) Depending on the nature of M and X and on the environment, the selenenate-κSe **2-MX**, the selenenate-κO **4-MX**, and the addition product **5-MX** can be formed.

In this work, we present the results of a computational mechanistic investigation of the reduction of H_2_O_2_ by PhSeMX (**1-MX** in Scheme 2, M = Zn, Cd, Hg; X= Cl, Br, I), elucidating the role of the metal as well as the effect of the halogen. Different scenarios are described: depending on the metal, the halogen, and the environment, we assist to the formation of the selenenate-κSe **2-MX**, the selenenate-κO **4-MX**, and the addition product **5-MX** (Scheme 2). Last, we describe the essential features of the whole catalytic cycle following H_2_O_2_ reduction, rationalizing the peculiar role of zinc.

## 2 Computational Methods

All DFT calculations were carried out with the Amsterdam Density Functional (ADF) program 2019.307 and the Amsterdam Modeling Suite (AMS) 2020.104 program.^45,46^ Zeroth-order regular approximation (ZORA) was employed to include scalar relativistic effects in the calculations, as recommended in the presence of heavy atoms.^47^ For all the geometry optimizations, the OLYP^48,49^ functional was used; this choice is supported by a benchmark study^50^ and analogous works on the oxidation of organochalcogenides.^44,51^ For all atoms, the TZ2P basis set was used, i.e., a large, uncontracted set of Slater-type orbitals of triple-ζ, augmented with two sets of polarization functions per atom; furthermore, a small frozen core approximation was employed. This level of theory is denoted as ZORA-OLYP/TZ2P. For all fully optimized structures, frequency calculations were performed to extract thermodynamic corrections and assess whether a true minimum was reached. All minima have real frequencies, whereas transition states have one imaginary frequency associated with the normal mode connecting reactants to products. Reaction paths were calculated using the intrinsic reaction coordinate (IRC) method,^52^ as implemented in AMS 2020. Implicit solvation effects (water) have been included, either in the optimization process (level of theory: COSMO-ZORA-OLYP/TZ2P) or in single-point energy calculations (level of theory: COSMO-ZORA-OLYP/TZ2P//ZORA-OLYP/TZ2P), by means of the conductor-like screening model (COSMO).^53^ To rationalize the energetics and trends of the oxidations, the activation strain model (ASM) was used, and energy decomposition analysis (EDA) was performed along the reaction coordinate^54−57^ using IRC profiles with the program PyFrag.^58^ ASM is a fragment-based approach that allows to express the total energy at any point along the reaction coordinate (ζ) as the sum of two contributions:
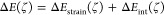
1where strain (Δ*E*_strain_) is the difference between the energy of the reactants with the structure they have at the investigated point and the energy of the free (undistorted) reactants and interaction (Δ*E*_int_) is the actual chemical interaction energy between the distorted reactants. The latter term can be further split into different chemically meaningful contributions within the EDA scheme:
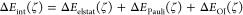
2where Δ*E*_elstat_(ζ) is the semiclassical electrostatic interaction between the unperturbed electron densities of the distorted fragments and Δ*E*_Pauli_(ζ) (Pauli or exchange repulsion) is related to the repulsion between occupied orbitals localized on the two fragments. Finally, Δ*E*_OI_(ζ) accounts for all of the occupied-void orbital interactions, such as the HOMO–LUMO interaction.

## 3 Results and Discussion

The oxidation of an organic chalcogenide is reported to lead to the corresponding chalcogenoxide^44^ even when a metal–chalcogen bond is involved.^59^ For dichalcogenides, a direct conversion between the chalcogenoxide and the corresponding anhydride, via the insertion of O in the chalcogen–chalcogen bond, is also possible although energetically disfavored.^51^ This chemical behavior can be modified by the presence of a selenium–metal bond; thus, the product of the reduction of H_2_O_2_ by **1-ZnCl**, i.e., the first step of the catalytic cycle shown in Scheme 1A, should not be taken for granted without accurate scrutiny. We started our investigation focusing on this reaction in the gas phase; then, the role of the metal center was evaluated by replacing Zn with its siblings of group 12 (Cd and Hg). The effect of the solvation (water) on the mechanism and energetics was evaluated; finally, the role of the halogen was assessed by replacing Cl with Br on the three compounds.

### 3.1 Role of the Metal

**Figure 1:**
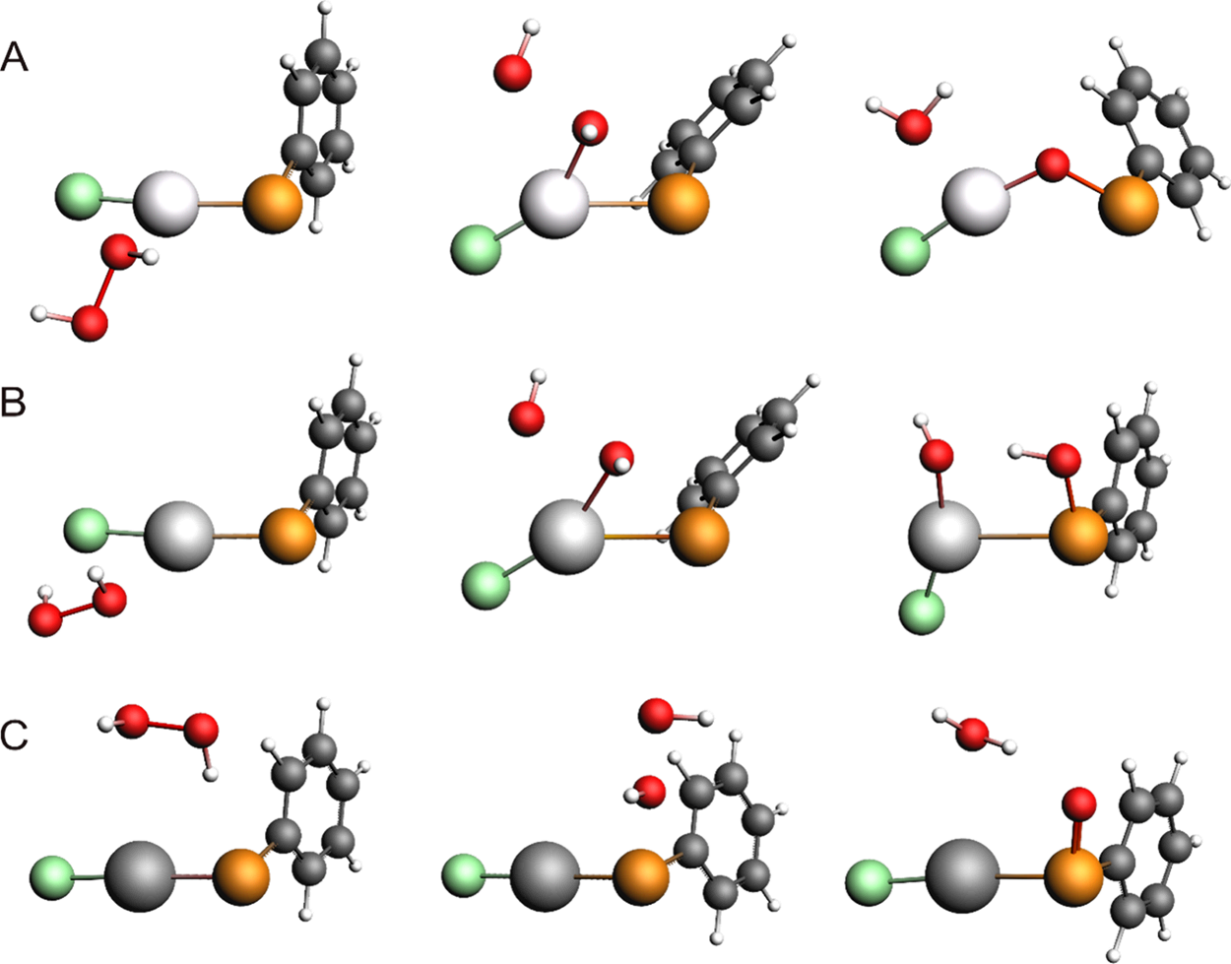
Fully optimized structures of the reactant complex (RC), transition state (TS), and product complex (PC) involved in the reduction of H_2_O_2_ by (A) **1-ZnCl**, (B) **1-CdCl**, and (C) **1-HgCl**. Level of theory: ZORA-OLYP/TZ2P.

**Figure 2:**
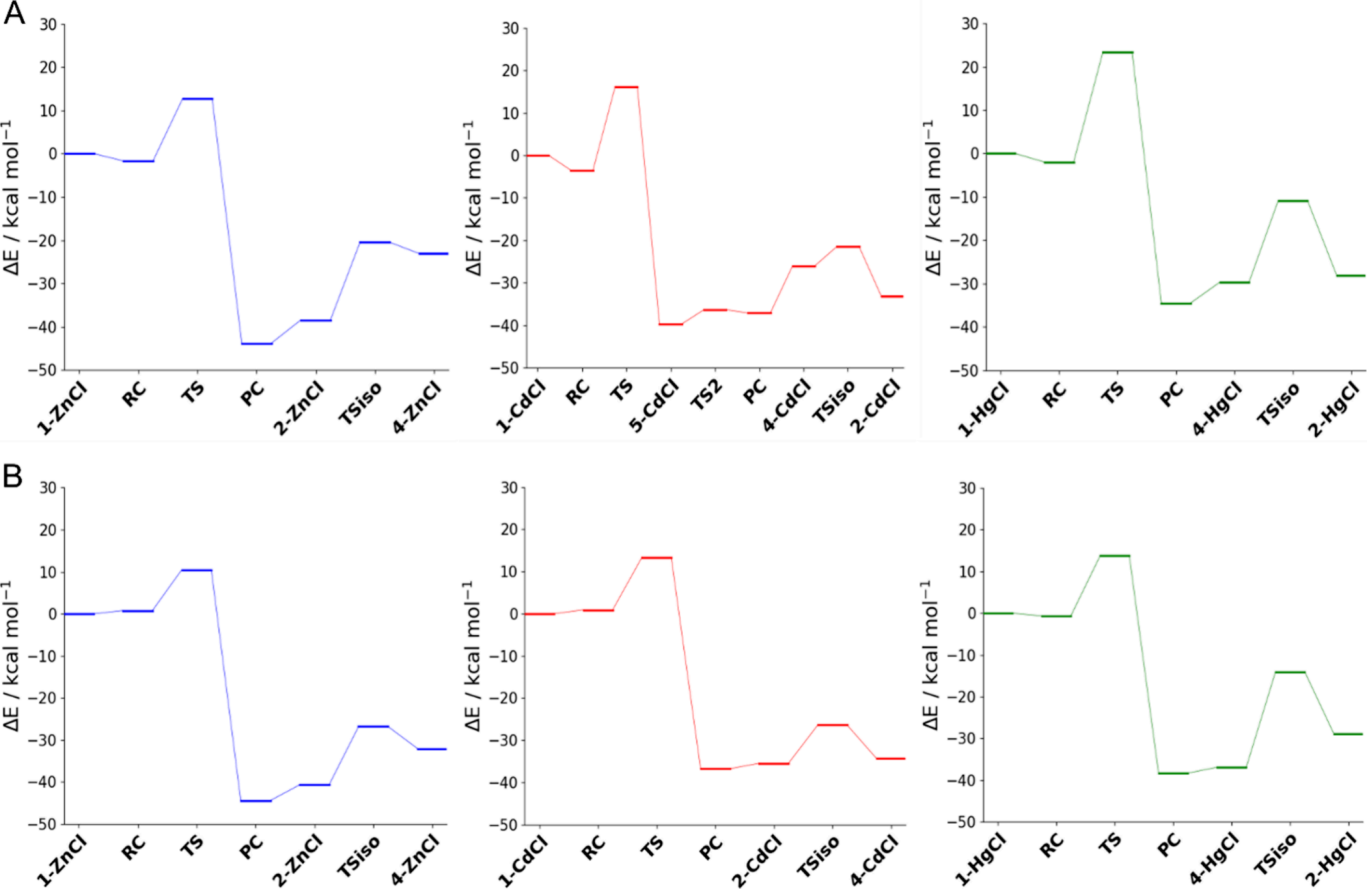
Energy profiles (kcal mol^–1^) relative to the free reactants for **1-ZnCl** (blue), **1-CdCl** (red), and **1-HgCl** (green) oxidation and isomerization in (A) gas phase and (B) water. For the isomerization, the energy of a single water molecule was added to the stationary points for consistency. Level of theory: (COSMO)-ZORA-OLYP/TZ2P.

In the gas phase, when the reactants approach, a reactant complex (RC) forms (Figures 1A and 2A), which is weakly stabilized with respect to the free reactants, i.e., **1-ZnCl** and H_2_O_2_. A product complex (PC), lying 42.14 kcal mol^–1^ below RC, is produced crossing a barrier of 14.39 kcal mol^–1^ (Table 1), in which a water molecule is loosely coordinated to the oxidized product with the oxygen atom inserted between Zn and Se (Figures 1A and 2A). Thus, DFT calculations (level of theory: ZORA-OLYP/TZ2P) fully support the mechanism proposed by Santi et al.:^38^ all attempts to find a transition state leading directly to **4-ZnCl** (Scheme 2) failed. This latter species, **4-ZnCl**, was located on the PES (potential energy surface), but it is destabilized by 15.35 kcal mol^–1^ with respect to **2-ZnCl**.

**Table 1:** Electronic Energies (kcal mol^–1^) Relative to the Free Reactants for the Reduction of H_2_O_2_ by **1-MCl** in the Gas Phase^a^

	RC	TS	PC	2-MCl	4-MCl
Zn	–1.68	12.71 (14.39)	–43.82	–38.44	–23.09
Cd	–3.49	16.18 (19.67)	–37.10^b^	–33.13	–26.04
Hg	–1.97	23.40 (25.37)	–34.47	–28.10	–29.67

a Activation energies relative to reactant complexes are given in parentheses. Level of theory: ZORA-OLYP/TZ2P.

b PC in this case is formed after the second TS with a small activation energy of 3.36 kcal mol^–1^.

To analyze the effect of the metal center on the reduction of H_2_O_2_, the same reaction was studied by replacing Zn in **1-ZnCl** with its heavier group 12 siblings (Cd and Hg) at the same level of theory (ZORA-OLYP/TZ2P). Cd- and Hg-substituted reactants (**1-CdCl** and **1-HgCl**) undergo the same reaction with H_2_O_2_, but the mechanism differs from metal to metal. **1-CdCl** oxidation shows a transition state quite similar to the one found for **1-ZnCl**, but the outcome of the reaction is different (Figure 1B). Starting from a much more stabilized RC (−3.49 kcal mol^–1^), the reaction proceeds with the attack of the chalcogen on one oxygen of the peroxide, O–O bond cleavage, and binding of the second OH fragment of H_2_O_2_ to the metal. Thus, we do not assist in the intramolecular proton transfer leading to the formation and exit of water. As a consequence, the addition product **5-CdCl** forms (Scheme 2 and Figure 2A). This species is characterized by two OH groups bonded to Cd and Se, respectively, and is stabilized by 36.22 kcal mol^–1^ with respect to the RC (Table 1). Thus, the reduction of H_2_O_2_ in the presence of Cd instead of Zn implies a higher activation energy (19.67 vs 14.39 kcal mol^–1^) and leads to a different product, which is less stabilized with respect to the corresponding RC.

Finally, H_2_O_2_ is reduced by **1-HgCl** that is directly oxidized to **4-HgCl** without appreciable perturbation of the metal–selenium bond (Scheme 2 and Figure 1C), as previously observed by some of us for the oxidation of analogous Hg compounds.^59^ Compared to the reduction by **1-ZnCl**, a higher activation energy is computed (25.37 vs 14.39 kcal mol^–1^), and the PC is much less stabilized with respect to the RC (−32.50 vs −42.14 kcal mol^–1^).

To better understand the behavior of **1-CdCl**, we made the hypothesis that the oxidation of **1-CdCl** to **5-CdCl** can be considered as the first step for the formation of **4-CdCl**. The addition product is a selenenic acid coordinated to a cadmium hydroxy halide; this structure suggests the possibility of a proton transfer from the Se-bonded OH moiety to the vicinal hydroxyl group with the cleavage of a water molecule from the metal center. This second step was confirmed by the presence of a transition state (TS2 in Figure 2A) with a modest energy barrier (3.36 kcal mol^–1^ with respect to **5-CdCl**). Notably, the product complex lies very close to the transition state, i.e., only 0.75 kcal mol^–1^ below. As final products, a water molecule and the selenenate-κSe **4-CdCl**, analogous to **4-HgCl**, are formed lying at −26.04 kcal mol^–1^ with respect to the initial free reagents.

**Figure 3:**
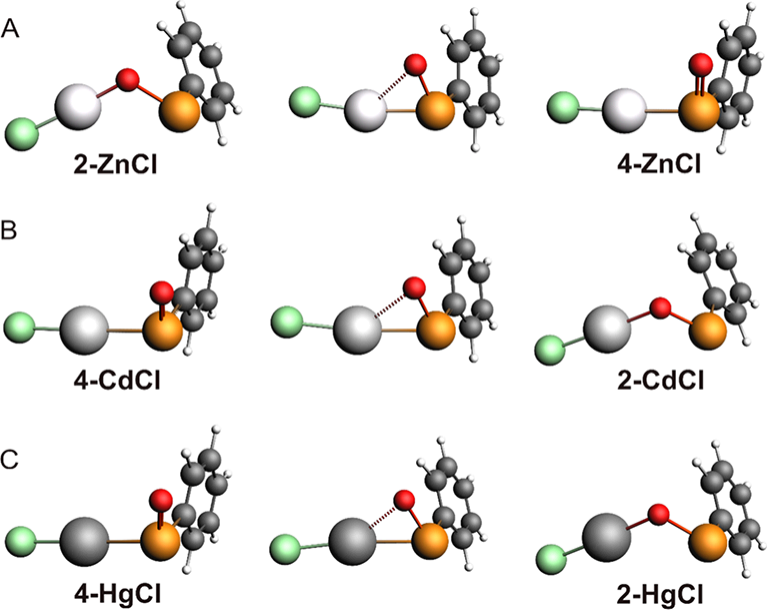
Fully optimized structures of the reactant, transition state (TS_iso_), and product involved in the isomerization of (A) **2-ZnCl**, (B) **4-CdCl**, and (C) **4-HgCl**. Level of theory: ZORA-OLYP/TZ2P.

In all cases shown in Figure 1, the reactions end with peroxide O–O bond breaking. Although **4-ZnCl** was located at a much higher energy than **2-ZnCl**, we searched for the transition state connecting them, and as expected, their conversion requires a rather high activation energy (17.97 kcal mol^–1^). Actually, also **4-CdCl** and **4-HgCl** might undergo isomerization to the selenenates-κSe **2-CdCl** and **2-HgCl**, respectively. In fact, we can hypothesize the existence of an additional step leading to a product analogous to **2-ZnCl**. We found that, for Cd, this step may occur easily, crossing a moderate energy barrier of 4.58 kcal mol^–1^ and accompanied by the release of 7.09 kcal mol^–1^. However, both **4-CdCl** and **2-CdCl** are less stable than **5-CdCl** (Figure 2A). In contrast, in the case of Hg, the isomerization of **4-HgCl** to **2-HgCl** seems difficult because of its relatively high activation energy (18.84 kcal mol^–1^) and a slight destabilization of the product by 1.57 kcal mol^–1^ (Figure 2A). In Figure 3, the structures of the reactants, the transition states, and the corresponding products of these isomerizations are shown.

The evidence that **5-MCl** is formed only in the case of Cd prompted us to seek if **5-ZnCl** and **5-HgCl** exist on the PES. **5-ZnCl** was found to lie 46.40 kcal mol^–1^ below **1-ZnCl** and, thus, 2.58 kcal mol^–1^ below the product complex of **2-ZnCl**. In contrast, **5-HgCl** lies 30.26 kcal mol^–1^ below **1-HgCl** and is less stable than the product complex of **4-HgCl**, which is directly formed, by 4.21 kcal mol^–1^.

However, to draw the correct conclusions about the relative stability of the different oxidation products, thermodynamic corrections must be added. Gibbs free energy values computed in the gas phase at 298 K and 1 atm are reported in Table 2. The inclusion of entropic effects discloses that any product complexes and the addition products **5-MCl** are destabilized on the PES, whereas free products **2-MCl** and **4-MCl** become the most stable species depending on the metal. In the case of Zn, **2-ZnCl** is more stable than **5-ZnCl** by 2.15 kcal mol^–1^, and **2-CdCl** is more stable than **5-CdCl** by 3.19 kcal mol^–1^. In the case of Hg, **5-HgCl** is destabilized with respect to **4-HgCl** by 11.89 kcal mol^–1^. These results confirm that **5-ZnCl** and **5-HgCl** can be ruled out from the oxidation mechanism in the presence of these metals. Thus, the predominant species formed in the oxidation of Zn and Cd reactant is the selenenate-κO, whereas in the oxidation of Hg reactant, it is the selenenate-κSe.

**Table 2:** Gibbs Free Energies (kcal mol^–1^) Relative to the Free Reactants for the Reduction of H_2_O_2_ by **1-MCl** in the Gas Phase^a^

	RC	TS	PC	2-MCl	4-MCl	5-MCl
Zn	7.58	20.13	–33.16	–38.66	–24.65	–36.51
Cd	7.29	24.05	–27.22^b^	–33.37	–27.58	–30.18
Hg	9.07	32.72	–24.52	–28.31	–29.72	–17.83

a Level of theory: ZORA-OLYP/TZ2P.

b PC in this case is formed after the second transition state.

The different oxidation paths make the quantitative comparison between the energetics not straightforward, particularly in the case of Cd. Qualitatively, considering the PCs, they all lie at a negative energy with respect to the corresponding RCs, and on going from Zn to Hg, the PCs are less and less stabilized. The same trend is observed for the most stable products formed, i.e., **2-ZnCl**, **2-CdCl**, and **4-HgCl**. The same trends are observed when the Gibbs free energies are considered (Table S1). Thus, we conclude that the reactions become less favored moving from Zn to Hg. Conversely, the **4-MCl** electronic as well as Gibbs free energies follow the opposite trend. This explains why **4-MCl** is the most stable product only for oxidation of the Hg compound. The activation energies (TS, Table 1 and Table S1) can also be easily rationalized: the values increase along group 12. Hence, **1-ZnCl** has the lowest activation energy.

**Figure 4:**
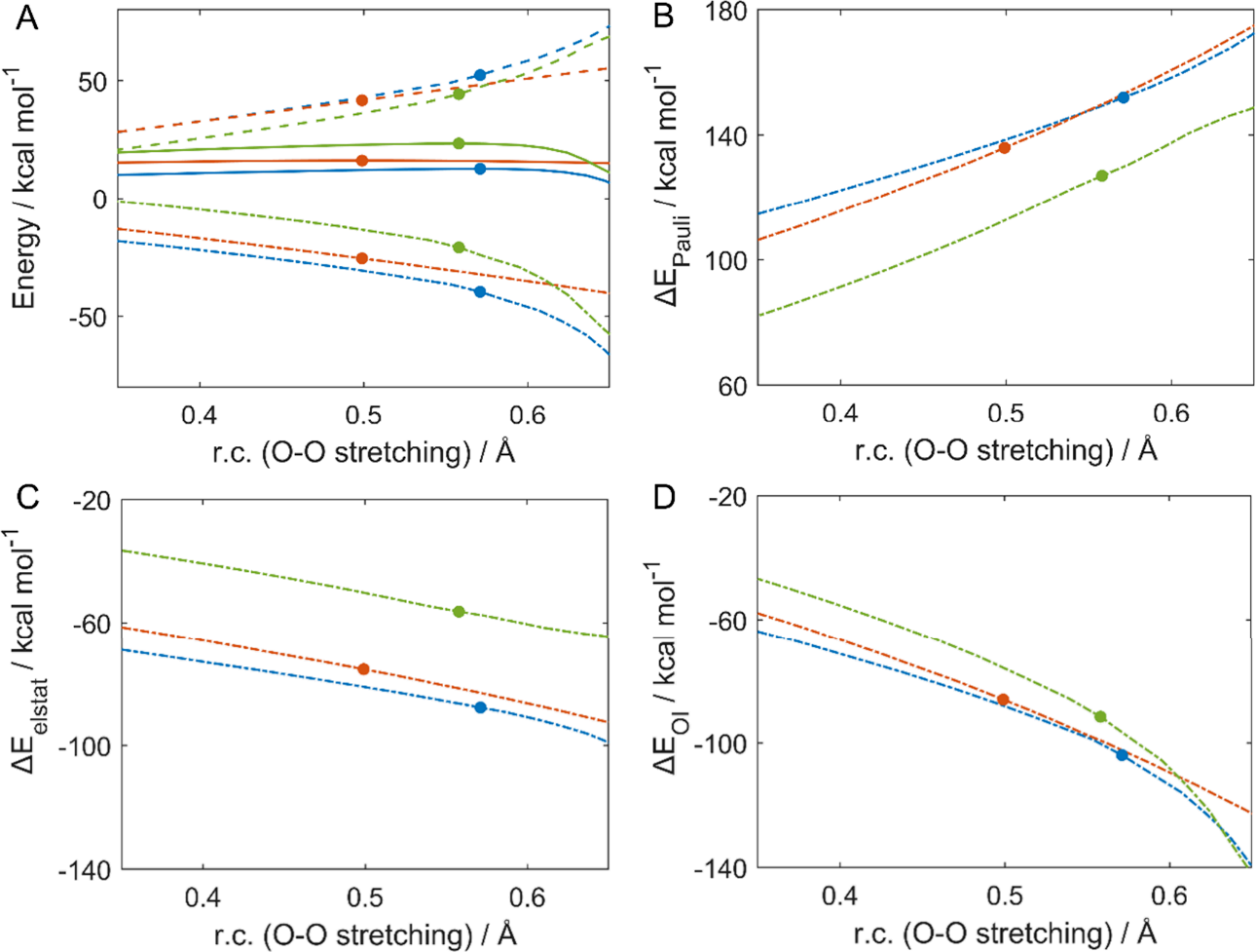
(A) Activation strain model of **1-ZnCl** (blue lines), **1-CdCl** (red lines), and **1-HgCl** (green lines) oxidations: energy profiles (solid lines), strain contributions (dashed lines), and interaction contributions (dash-dotted lines). Energy decomposition analysis: (B) Pauli repulsion, (C) electrostatic interaction, and (D) orbital interaction. The position of the transition states is indicated by filled dots. The reaction coordinate is defined as r.c.= (d_O–O_-d_O–O_^0^), where d_O–O_^0^ represents the O–O bond length in the reactant complex of each reaction. Level of theory: ZORA-OLYP/TZ2P.

The reduction of H_2_O_2_ by **1-MCl** (M = Zn, Cd, and Hg) was analyzed in the frame of the activation strain model (see Computational Methods). The chosen reaction coordinate (r.c.) is the O–O distance that changes upon bond breaking because it is the molecular event common to all three different processes. As shown in Figure 4A, an identical strain along the reaction coordinate characterizes the Zn and Cd systems; this stems from the similarity of the structures of their transition states. In contrast, a much lower Δ*E*_strain_ is computed for the Hg systems because, in this case, the overall molecular structure remains almost unperturbed. Nevertheless, the activation energy decreases from that of **1-HgCl** to **1-ZnCl**. This evidence reveals the controlling role of Δ*E*_*i*nt_: the Zn system has the most stabilizing interaction, very close to the Cd one, which decreases (in absolute value) for the Hg system. The relative position of the transition states along the reaction coordinate (Figure 4A) is more difficult to rationalize; the earliest one is found for the Cd system followed by Hg and Zn in close proximity. The absence of a trend reflecting the group sequence is likely due to the differences in the reaction mechanisms. This holds especially true for **1-CdCl**: no water molecule is cleaved during the oxidation, and almost no change in the slope of strain/interaction curves is observed. Furthermore, the transition state of the Hg system is a bit earlier than the Zn one because of the significantly lower strain computed for the former along the reaction coordinate.

Because Δ*E*_int_ is mainly responsible for the activation energy trend, through EDA, the prevailing component can be assessed. Both the electrostatic interaction (Figure 4C) and the orbital interaction (Figure 4D) vary consistently with the interaction energy, whereas Pauli repulsion (Figure 4B) is characterized by a reverse energy order of the curves; i.e., **1-ZnCl** is the most destabilized, and **1-HgCl** is the least destabilized. The electrostatic interaction is the dominant contribution.

**Figure 5:**
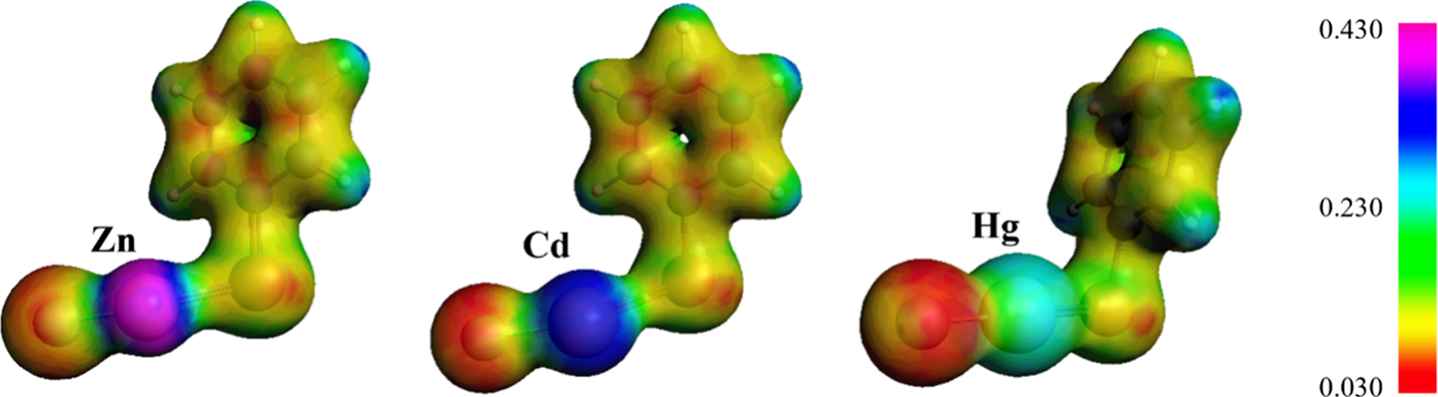
Electrostatic potential maps of **1-MCl** at r.c. = 0.5. SCF Coulomb potential was computed in atomic units. Level of theory: ZORA-OLYP/TZ2P.

These trends can be rationalized by considering the size (atom radius) of the metal centers. Zn is the smallest metal; thus, its orbital overlap with oxygen is better than that of its heavier siblings. Indeed, the orbital contributions involve both the metal and selenium as acceptors in the case of Zn and Cd, whereas for Hg, only the lone pair of Se is involved (Figure S1). In addition, Pauli repulsion is larger in **1-ZnCl** than in **1-CdCl** and **1-HgCl**; in fact, the repulsion between occupied orbitals is higher in smaller metals. Finally, **1-ZnCl** is characterized by the highest electrostatic potential density. **1-HgCl** represents the opposite extreme, and **1-CdCl** has intermediate properties. These outcomes are supported by the electrostatic potential maps (Figure 5).

### 3.2 Solvation Effect

The introduction of water solvation in the calculations does not modify the reduction mechanism of H_2_O_2_ by **1-ZnCl**; conversely, in the case of **1-CdCl**, changes are foreseen. The mechanisms for all three reagents were studied, including the solvation in the geometry optimization of stationary points. Figure 2B shows the energy profiles connecting the free reactants to the corresponding selenenate-κSe **2-MCl** and selenenate-κO **4-MCl** in water. The reduction of H_2_O_2_ by **1-ZnCl** is still a single step process leading to the formation of water and **2-ZnCl**; the isomerization of the latter to **4-ZnCl** remains energetically disfavored. Differently from the results in the gas phase commented in the previous paragraph, the reduction mechanism of H_2_O_2_ by **1-CdCl** in water is similar to the Zn one: the reagent is directly oxidized to **2-CdCl**, which is slightly more stable than **4-CdCl**. Finally, the reduction of H_2_O_2_ by **1-HgCl** in water retains the same mechanism predicted in the gas phase, i.e., a single step formation of water and **4-HgCl**; the isomerization of the latter to **2-HgCl** is energetically disfavored.

Table 3 shows the electronic energies (kcal mol^–1^) of the stationary points located on the PESs of these reactions in water (level of theory: COSMO-ZORA-OLYP/TZ2P) relative to the free reactants. The main energy trends found in the gas phase are maintained also in water: the energies of **2-MCl** are still more negative in the presence of the lighter metals, whereas the energies of **4-MCl** still follow the opposite trend. It is noteworthy that solvation stabilizes both products for the three reactants, but particularly **4-MCl**. In the case of Zn, the better stabilization of **4-ZnCl** than that of **2-ZnCl** is not sufficient to invert the energy order of the two possible products; thus, the isomerization to the selenenate-κSe is still energetically disfavored. Analogous considerations can be drawn in the case of Cd: **2-CdCl** is still more stable than **4-CdCl**, but the energy difference in water is just 1.17 kcal mol^–1^, suggesting that they can both be thermodynamically available. Finally, for reduction of H_2_O_2_ by **1-HgCl**, the most stable product remains **4-HgCl**, and the isomerization to **2-HgCl** becomes even more energetically disfavored. It is also noteworthy that in water, the direct oxidation of **1-HgCl** to **4-HgCl** becomes energetically more favored than the direct oxidation of **1-CdCl** to **2-CdCl**_,_ due to the different stabilization of the two products.

**Table 3:** Electronic Energies (kcal mol^–1^) Relative to the Free Reactants for the Reduction of H_2_O_2_ by **1-MCl** in Water^a^

	RC	TS	PC	2-MCl	4-MCl
Zn	0.77	10.51 (9.74)	–44.33	–40.67	–32.04
Cd	0.90	13.37 (12.47)	–36.76	–35.47	–34.30
Hg	–0.64	13.73 (14.37)	–38.31	–28.94	–36.99

a Activation energies relative to reactant complexes are given in parentheses. Level of theory: COSMO-ZORA-OLYP/TZ2P.

Kinetically, the reduction of H_2_O_2_ by **1-ZnCl** is the most favored reaction, whereas the activation energies for **1-CdCl** and **1-HgCl** in water are higher and comparable. This can be mainly ascribed to the stabilization of the transition state in the case of the Hg reactant, whose geometry is neatly different from those with Zn and Cd, which remain very similar to each other, as in the gas phase. In particular, in the transition state involving Hg, the oxygen atom of the peroxide is close to Se (distances Hg–O and Se–O are 3.8 and 2.3 Å, respectively), whereas in the cases of Zn and Cd, the analogous oxygen is close both to the metal and to Se (distances Zn–O and Cd–O are 2.0 and 2.4 Å, whereas distance Se–O is 2.5 and 2.4 Å for the former and the latter system, respectively). Thus, in the former case, the peroxide is more exposed to the polar environment, explaining the higher level of stabilization of the heaviest metal TS in water. However, because of a concomitant slight stabilization of the Hg reactant complex and destabilization of the Cd reactant complex, the energy barriers relative to these intermediates in the presence of the heaviest metals are no longer equal; thus, the activation energy trend, with respect to the reactant complexes, is restored.

As in the gas phase, Gibbs free energy values (Table S1) show that the product complexes and the addition product **5-MCl** is destabilized on the PES (**5-HgCl** does not even exist at the employed level of theory). Overall, the predominant species formed in the oxidation of Zn and Cd reactants is the selenenate-κO, whereas in the oxidation of Hg reactant, it is the selenenate-κSe, analogously to the gas phase.

### 3.3 Effect of the Halogen

The bromo-derivative of **1-ZnCl** was also synthesized.^1^ Thus, to assess the role of the halogen, the reduction of H_2_O_2_ was studied in the gas phase for **1-MBr** too. The presence of Br does not affect the mechanism: **1-ZnBr** is directly oxidized to **2-ZnBr** with an activation energy of 14.01 kcal mol^–1^, releasing 41.56 kcal mol^–1^; **4-ZnBr** is destabilized by 15.16 kcal mol^–1^ with respect to **2-ZnBr**. Conversely, the reduction mechanism of H_2_O_2_ by **1-CdBr** in the gas phase changes: water and **2-CdBr** are directly formed in a single step, similarly to what we observe for **1-CdCl** in water. Furthermore, **2-CdBr** remains more stable than **4-CdBr**; thus, isomerization is energetically disfavored. Finally, also the reduction mechanism of H_2_O_2_ by **1-HgBr** is not affected by the presence of a different halogen; water and **4-HgBr** are formed in a single step, and like for the Cl derivative, **2-HgBr** lies at higher energy on the PES. The energy profiles of the reduction of H_2_O_2_ by **1-MBr** in the gas phase are reported in Figure S2A, and the data are shown in Table 4.

**Table 4:** Electronic Energies (kcal mol^–1^) Relative to the Free Reactants for the Reduction of H_2_O_2_ by **1-MBr** in the Gas Phase^a^

	RC	TS	PC	2-MBr	4-MBr
Zn	–1.48	12.53 (14.01)	–41.56	–38.55	–23.39
Cd	–2.80	15.82 (18.62)	–38.23	–33.40	–26.22
Hg	–1.68	23.10 (24.78)	–34.56	–28.40	–29.98

a Activation energies relative to reactant complexes are given in parentheses. Level of theory: ZORA-OLYP/TZ2P.

Overall, the presence of Br does not modify the trends on the reaction and activation energies stated for **1-MCl**. **1-ZnBr** still has the lowest energy barrier and the most favored energetics for the oxidation to **2-ZnBr**, whereas the **1-HgBr** case lies at the opposite extreme: the highest energy barrier and the most favored energetics are computed for a process leading to the formation of **4-HgBr**. We can conclude that the replacement of the halogen has a negligible effect even on the relative stationary points’ energies; indeed, the energy values in Tables 1 and 4 show no significant differences.

Notably, the reduction mechanism of H_2_O_2_ by **1-MBr** does not include the formation of any **5-MBr** intermediate, not even for Cd. However, these three compounds were studied for completeness. **5-ZnBr** and **5-CdBr** lie at −46.42 and −39.87 kcal mol^–1^ with respect to the free reactants, respectively. Hence, they are the most stable products on the basis of electronic energies. Conversely, **5-HgBr** is not the most stable compound if compared to the product complex of **4-HgBr** (−30.35 vs −34.56 kcal mol^–1^).

When thermodynamic corrections are added and Gibbs free energies are considered (Table S2), this picture changes, and **5-MBr** is in all cases destabilized on the PES; thus, **2-ZnBr** becomes more stable than **5-ZnBr** by 2.20 kcal mol^–1^, and **2-CdBr** becomes more stable than **5-CdBr** by 4.67 kcal mol^–1^. Lastly, **5-HgBr**, which was not the most stable compound even without including thermodynamic corrections, is further destabilized with respect to **4-HgBr** by 12.30 kcal mol^–1^; notably, the same observations hold true for **5-MCl** for each different metal.

To further investigate the reduction of H_2_O_2_ by **1-MBr**, ASM/EDA was performed using the IRC geometries computed along the chosen reaction coordinate (O–O distance). As expected, the results (Figure S3) are fully consistent with the analysis on **1-MCl**. Equal strain contributions are obtained for **1-ZnBr** and **1-CdBr** until the transition state, whereas the strain of **1-HgBr** is significantly lower. The activation energy trend is still reproduced by the interaction contributions and particularly by the electrostatic and orbital interactions. It is remarkable that the oxidations of **1-CdX** show no difference in the analysis despite leading to different products. This can be explained because the two reactions proceed with the same mechanism until they reach a similar transition state; then, **1-CdCl** evolves to **5-CdCl**, whereas **1-CdBr** evolves directly to **2-CdBr**.

Finally, the solvation effects were also considered for the reduction of H_2_O_2_ by **1-MBr**. In this case, the presence of water does not modify the mechanisms for any reactant, which remains a single step leading to the formation of water and **2-ZnBr**, **2-CdBr**, or **4-HgBr**, respectively. Hence, the mechanism was studied by performing single-point energy calculations in water using the geometry optimized in the gas phase (level of theory: COSMO-ZORA-OLYP/TZ2P//ZORA-OLYP/TZ2P). The energy profiles of these reactions are reported in Figure S2B. Table 5 shows the electronic energies (kcal mol^–1^) of the stationary points in water. Similarly to the reduction of H_2_O_2_ by **1-MCl**, the solvation stabilizes **4-MBr** more than **2-MBr**, leading to the same outcomes: for Zn, **2-ZnBr** remains more stable; for Cd, **2-CdBr** is slightly more stable; finally, for Hg, **4-HgBr** becomes much more stable than **2-HgBr**. Still, when the solvent is included, the direct oxidation to **2-CdBr** became slightly less energetically favored than the direct oxidation to **4-HgBr.**

**Table 5:** Electronic Energies (kcal mol^–1^) Relative to the Free Reactants for the Reduction of H_2_O_2_ by **1-MBr** in Water^a^

	RC	TS	PC	2-MBr	4-MBr
Zn	1.03	10.76 (9.73)	–41.82	–41.20	–32.42
Cd	0.88	16.02 (15.14)	–36.16	–36.22	–34.56
Hg	–1.04	12.57 (13.61)	–36.67	–29.96	–37.33

a Activation energies relative to reactant complexes are given in parentheses. Level of theory: COSMO-ZORA-OLYP/TZ2P//ZORA-OLYP/TZ2P.

The activation energy trend in the gas phase is lost in water: the lowest barrier is still found in the presence of Zn, but the reduction of H_2_O_2_ by **1-CdBr** is the kinetically most difficult case. Differently from the reduction of H_2_O_2_ by **1-MCl** in water, the trends are maintained also when the energy barriers are computed with respect to the reactant complexes. The loss of a regular kinetic trend along the group is still ascribed to the greater stabilization of the transition state in water in the case of Hg. Indeed, the presence of Cl or Br does not significantly alter the geometry structures of reactants and transition states. Gibbs free energies (Table S3) show analogous thermodynamic and kinetic data, but the energy difference between **2-CdBr** and **4-CdBr** is close to zero; i.e., they are isoenergetic.

Lastly, the halogen series was completed by the inclusion of iodine in the analyzed systems (Table S4). No halogen effect was found because the iododerivatives show no significant difference in either the mechanism or the energetics when compared to the reduction of H_2_O_2_ by **1-MBr**.

### 3.4 Beyond Oxidation

**Scheme 3:**
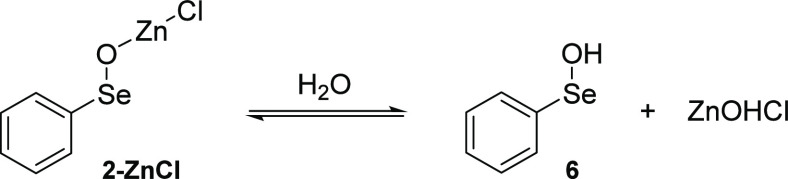
Hydration Equilibrium of **2-ZnCl** with the Formation of Phenyl Selenenic Acid **6** and ZnOHCl

According to Scheme 1A, after the reduction of H_2_O_2_ by **1-ZnCl**, some other steps are necessary to complete the catalytic cycle. The first one is the reaction with glutathione (GSH) leading to a selenosulfide, which is a reasonable product if the hydration equilibrium of **2-ZnCl** is considered (Scheme 3). According to this equilibrium, the phenyl selenenic acid **6** can be involved in the catalytic cycle. The labile nature of selenenic acids is well-known in the literature;^60−64^ indeed, it can readily react with thiols, like GSH, and selenols with the formation of a chalcogen–chalcogen bond, as in the GPx cycle^39,40^ (Scheme 1B). Hence, the hydration equilibria in the presence of the different metals (Zn, Cd, Hg) and halogens (Cl, Br) have been studied, starting from the most stable products for each metal compound, i.e., **2-ZnX**, **2-CdX**, and **4-HgX**.

Electronic reaction energies, computed in both the gas phase and water, are shown in Table 6. Regardless of the halogen and the environment, the reaction energies of **2-ZnX** are close to zero; therefore, **6** can actually form in equilibrium with **2-ZnCl**. When going to Cd and Hg, the reaction energies become more positive; the formation of **6** can still occur in a small percentage in the equilibria of **2-CdX**. Conversely, when **4-HgX** is considered, the hydration becomes neatly disfavored, especially when the water environment is included. This is consistent with the previous results because **4-MX** is largely stabilized in water and, thus, their reactivity decreases. Therefore, the formation of compound **6** seems to be limited to the hydration of compounds **2-ZnX** and **2-CdX**. Gibbs free reaction energies (Table S5) also support this conclusion because the hydration of **2-ZnX** and that of **2-CdX** are both exergonic (close to zero for Cd), whereas the reaction is endergonic for **4-HgX**. Although a small quantity of **6** is sufficient to proceed in the catalytic cycle, from now on, only the reactivity of **1-ZnX** will be discussed because they were successfully synthesized and they are associated with the smallest activation barriers for the first step of the cycle, i.e., the hydroperoxide reduction.

**Table 6:** Electronic Reaction Energies (kcal mol^–1^) for the Hydration Equilibria of **2-ZnX**, **2-CdX**, and **4-HgX** in the Gas Phase and in Water^a^

	Δ*E*_**gas phase**_			Δ*E*_water_		
	Zn	Cd	Hg	Zn	Cd	Hg
Cl	0.05	1.55	3.35	–0.16	1.14	9.09
Br	0.18	1.73	3.69	0.03	1.32	9.02

a Levels of theory: ZORA-OLYP/TZ2P and COSMO-ZORA-OLYP/TZ2P//ZORA-OLYP/TZ2P.

Although significant, the formation of **6** is not sufficient to explain the catalytic activity of **1-ZnCl** that, as reported, is not just a precursor of a selenenic acid;^38^ indeed, the Zn center, as a Lewis acid, may play an active role. After its formation, **6** can react with GSH (which can be modeled as methyl thiol, MeSH, as chosen in previous studies^65−69^) forming a selenosulfide; then, a second molecule of GSH/MeSH can in principle attack either the S or the Se center, but only the first event leads to the recovery of the initial catalyst. This is the most delicate step for designing efficient low-molecular-weight GPx mimic molecules,^70^ and in this framework, the action of the Zn center can be important to drive the nucleophilic attack.

**Scheme 4:**
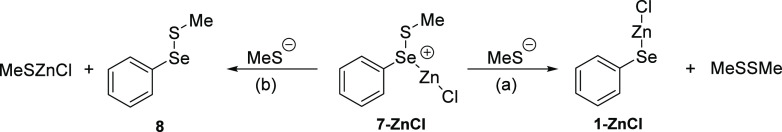
Competitive Reactions between **7-ZnCl** and MeS^–^ Path (a) involves sulfur on **7-ZnCl** leading to the recovery of the catalyst **1-ZnCl** and closing the cycle. Path (b) involves selenium on **7-ZnCl** leading to the selenosulfide **8** and thus resulting in a scrambling process.

In the last part of the GPx-like mechanism of Santi’s reagent, different paths can be foreseen. The postulated coordination of the Zn center to the Se atom of the selenosulfide **8** leads to **7-ZnCl**, and an alkaline environment is generated (analogously to the second step in Scheme 1A). In these conditions, MeSH can be deprotonated, thus increasing the nucleophilicity of its sulfur center and thus its reactivity.^71^ Hence, the thiolate can react with **7-ZnCl** either at the S site (path a), leading to the recovery of the catalyst **1-ZnCl** and closing the cycle, or at the Se site (path b), leading again to selenosulfide **8** (Scheme 4). The same products of path b can also be obtained if the attack occurs at the Zn center because the nucleophile is the same as the previous step, i.e., glutathione anion. The energetics of these competitive reactions were investigated *in silico* modeling GS^–^ as a methyl thiolate.

Electronic reaction energies were computed in water (level of theory: COSMO-ZORA-OLYP/TZ2P). For paths (a) and (b), they are −40.19 and −39.06 kcal mol^–1^, respectively. Thus, path (a) seems to be energetically slightly more favored. Similar results were obtained by replacing the halogen, i.e., Br instead of Cl: the reaction energies become −39.72 (a) and −38.49 kcal mol^–1^ (b), respectively. Moreover, if the Zn center had no active role in this step of the catalysis, reaction (a) would lead to a phenyl selenolate PhSe^–^ with Δ*E*= −7.11 kcal mol^–1^. Thus, the Zn center may indeed play a thermodynamic role as demonstrated by the more negative reaction energy in the presence of the metal. Furthermore, the presence of a Zn^2+^ ion might also have a kinetic effect from both an electronic and steric point of view; the reduced electrophilicity of Se and the increased steric hindrance around this atom may disfavor path (b). These aspects require a thorough systematic analysis that is out of the purpose of this work.

## 4 Conclusions

To sum up, our study paves the way for the complete understanding of the GPx-like mechanism of **1-ZnX**, focusing on the reduction of H_2_O_2_, which is the first and most peculiar step of the catalytic cycle. Furthermore, we have elucidated the role of the central metal by replacing Zn with its siblings in group 12, i.e., Cd and Hg. The reduction of H_2_O_2_ by **1-ZnX** was found to be favored both thermodynamically and kinetically; this is attributed to more favorable electrostatic and orbital interactions quantified in the framework of the ASM/EDA approaches. Moreover, the role of the halogen was investigated by systematically replacing Cl with Br; the reduction of H_2_O_2_ by the three metal compounds shows features very similar to those of both halogens. Finally, the inclusion of an implicit solvation has two main effects: (i) In water, the selenenate-κO **4-MX** is much more stabilized than the selenolate-κSe **2-MX**. However, this effect does not modify the main outcomes of the reduction of H_2_O_2_ by the three metal compounds. (ii) When **1-HgX** is involved in the reaction, the transition state is particularly stabilized, making the energy barrier of the process comparable to or even lower than the one associated with **1-CdX**.

For what concerns the last part of the GPx-like mechanism of Santi’s reagent, further studies are needed to quantitatively assess the kinetic effect of the Zn center considering all mechanistic possibilities because, as above-described, these reactions can occur at the S, Se, and Zn sites. Particularly, in this last case, multiple mechanisms should be considered, i.e., associative, dissociative, and interchange.

## Supporting Information Available

Gibbs free energies; additional energy profiles; additional ASM and EDA calculations; Cartesian coordinates, electronic energies, and imaginary frequencies of the investigated compounds (PDF)

## References

[ref1] Santi C.; Santoro S.; Battistelli B.; Testaferri L.; Tiecco M. Preparation of the First Bench-Stable Phenyl Selenolate: An Interesting “On Water” Nucleophilic Reagent. Eur. J. Org. Chem. 2008, 2008 (32), 5387–5390. 10.1002/ejoc.200800869.

[ref2] Santi C.; Capoccia L.; Monti B. Zinc-Selenium Reagents in Organic Synthesis. Phys. Sci. Rev. 2018, 3 (12), 20170129 10.1515/psr-2017-0129.

[ref3] Santi C.; Scimmi C. Selenium and Tellurium Complexes in Organic Synthesis. In Comprehensive Organometallic Chemistry IV; Elsevier: 2022; Vol. 11, pp 536–562. 10.1016/B978-0-12-820206-7.00082-2.

[ref4] Jardim G. A. M.; Bozzi Í. A. O.; Oliveira W. X. C.; Mesquita-Rodrigues C.; Menna-Barreto R. F. S.; Kumar R. A.; Gravel E.; Doris E.; Braga A. L.; da Silva Júnior E. N. Copper Complexes and Carbon Nanotube–Copper Ferrite-Catalyzed Benzenoid A-Ring Selenation of Quinones: An Efficient Method for the Synthesis of Trypanocidal Agents. New J. Chem. 2019, 43 (35), 13751–13763. 10.1039/C9NJ02026H.

[ref5] Huang X.; Xu X.-H. A New Method for the Synthesis of Allyl Arylselenides via the Reaction of the Zinc Allyl Selenoates with Diaryliodonium Salts. Synth. Commun. 1998, 28 (5), 801–805. 10.1080/00032719808006476.

[ref6] Huang X.; Xu X.-H. Insertion of Elemental Selenium into Zinc Carbon Bond and Application in Synthesis of α-Selenocarbonyl Compound. Synth. Commun. 1998, 28 (5), 807–811. 10.1080/00032719808006477.

[ref7] Santoro S.; Battistelli B.; Testaferri L.; Tiecco M.; Santi C. Vinylic Substitutions Promoted by PhSeZnCl: Synthetic and Theoretical Investigations. Eur. J. Org. Chem. 2009, 2009 (29), 4921–4925. 10.1002/ejoc.200900800.

[ref8] Salman S.; Schwab R.; Alberto E.; Vargas J.; Dornelles L.; Rodrigues O.; Braga A. Efficient Ring Opening of Protected and Unprotected Aziridines Promoted by Stable Zinc Selenolate in Ionic Liquid. Synlett 2011, 2011 (01), 69–72. 10.1055/s-0030-1259082.

[ref9] Jastrzebska; Mellea; Salerno; Grzes; Siergiejczyk; Niemirowicz-Laskowska; Bucki; Monti; Santi PhSeZnCl in the Synthesis of Steroidal β-Hydroxy-Phenylselenides Having Antibacterial Activity. Int. J. Mol. Sci. 2019, 20 (9), 2121 10.3390/ijms20092121. 31032813 PMC6539910

[ref10] Jastrzebska I.; Grzes P. A.; Niemirowicz-Laskowska K.; Car H. Selenosteroids - Promising Hybrid Compounds with Pleiotropic Biological Activity: Synthesis and Biological Aspects. J. Steroid Biochem. Mol. Biol. 2021, 213, 105975 10.1016/j.jsbmb.2021.105975. 34418527

[ref11] Jiang H.; Pan X.; Li N.; Zhang Z.; Zhu J.; Zhu X. Selenide-Containing High Refractive Index Polymer Material with Adjustable Refractive Index and Abbe’s Number. React. Funct. Polym. 2017, 111, 1–6. 10.1016/j.reactfunctpolym.2016.12.007.

[ref12] Kim Y.; Mulay S. V.; Choi M.; Yu S. B.; Jon S.; Churchill D. G. Exceptional Time Response, Stability and Selectivity in Doubly-Activated Phenyl Selenium-Based Glutathione-Selective Platform. Chem. Sci. 2015, 6 (10), 5435–5439. 10.1039/C5SC02090E. 28757944 PMC5510528

[ref13] Perin G.; Alves D.; Jacob R. G.; Barcellos A. M.; Soares L. K.; Lenardão E. J. Synthesis of Organochalcogen Compounds Using Non-Conventional Reaction Media. ChemistrySelect 2016, 1 (2), 205–258. 10.1002/slct.201500031.

[ref14] Azeredo J. B.; Penteado F.; Nascimento V.; Sancineto L.; Braga A. L.; Lenardao E. J.; Santi C. Green Is the Color”: An Update on Ecofriendly Aspects of Organoselenium Chemistry. Molecules 2022, 27 (5), 1597 10.3390/molecules27051597. 35268698 PMC8911681

[ref15] Tanini D.; Capperucci A.; Menichetti S. Nucleophilic Chalcogen-Containing Reagents. In Chalcogen Chemistry: Fundamentals and Applications; Lippolis V., Santi C., Lenardão E. J., Braga A. L., Eds.; The Royal Society of Chemistry: Croydon, 2023; pp 300–333. 10.1039/BK9781839167386-00300.

[ref16] Reich H. J. Functional Group Manipulation Using Organoselenium Reagents. Acc. Chem. Res. 1979, 12 (1), 22–30. 10.1021/ar50133a004.

[ref17] Mugesh G.; Singh H. B. Heteroatom-Directed Aromatic Lithiation: A Versatile Route to the Synthesis of Organochalcogen (Se, Te) Compounds. Acc. Chem. Res. 2002, 35 (4), 226–236. 10.1021/ar010091k. 11955051

[ref18] Liotta D. New Organoselenium Methodology. Acc. Chem. Res. 1984, 17 (1), 28–34. 10.1021/ar00097a005.

[ref19] Back T. G. Organoselenium Chemistry: A Practical Approach; Oxford Universitary Press: Oxford, 1999.

[ref20] Kumar A. V.; Reddy V. P.; Reddy C. S.; Rao K. R. Potassium Selenocyanate as an Efficient Selenium Source in C–Se Cross-Coupling Catalyzed by Copper Iodide in Water. Tetrahedron Lett. 2011, 52 (31), 3978–3981. 10.1016/j.tetlet.2011.05.068.

[ref21] Sridhar R.; Srinivas B.; Surendra K.; Krishnaveni N. S.; Rao K. R. Synthesis of β-Hydroxy Selenides Using Benzeneselenol and Oxiranes under Supramolecular Catalysis in the Presence of β-Cyclodextrin in Water. Tetrahedron Lett. 2005, 46 (51), 8837–8839. 10.1016/j.tetlet.2005.10.094.

[ref22] Saha A.; Saha D.; Ranu B. C. Copper Nano-Catalyst: Sustainable Phenyl-Selenylation of Aryl Iodides and Vinyl Bromides in Water under Ligand Free Conditions. Org. Biomol. Chem. 2009, 7 (8), 1652 10.1039/b819137a. 19343253

[ref23] Zhao H.; Hao W.; Xi Z.; Cai M. Palladium-Catalyzed Cross-Coupling of PhSeSnBu3 with Aryl and Alkyl Halides in Ionic Liquids: A Practical Synthetic Method of Diorganyl Selenides. New J. Chem. 2011, 35 (11), 2661 10.1039/c1nj20514e.

[ref24] Narayanaperumal S.; Alberto E. E.; de Andrade F. M.; Lenardão E. J.; Taube P. S.; Braga A. L. Ionic Liquid: An Efficient and Recyclable Medium for Synthesis of Unsymmetrical Diorganyl Selenides Promoted by InI. Org. Biomol. Chem. 2009, 7 (22), 4647 10.1039/b910699e. 19865700

[ref25] Gonçalves L. C.; Fiss G. F.; Perin G.; Alves D.; Jacob R. G.; Lenardão E. J. Glycerol as a Promoting Medium for Cross-Coupling Reactions of Diaryl Diselenides with Vinyl Bromides. Tetrahedron Lett. 2010, 51 (51), 6772–6775. 10.1016/j.tetlet.2010.10.107.

[ref26] Ricordi V. G.; Freitas C. S.; Perin G.; Lenardão E. J.; Jacob R. G.; Savegnago L.; Alves D. Glycerol as a Recyclable Solvent for Copper-Catalyzed Cross-Coupling Reactions of Diaryl Diselenides with Aryl Boronic Acids. Green Chem. 2012, 14 (4), 1030 10.1039/c2gc16427b.

[ref27] Mukherjee N.; Chatterjee T.; Ranu B. C. Reaction under Ball-Milling: Solvent-, Ligand-, and Metal-Free Synthesis of Unsymmetrical Diaryl Chalcogenides. J. Org. Chem. 2013, 78 (21), 11110–11114. 10.1021/jo402071b. 24116379

[ref28] Ananikov V. P.; Beletskaya I. P. Palladium-Catalyzed Addition of Disulfides and Diselenides to Alkynes under Solvent Free Conditions. Org. Biomol. Chem. 2004, 2 (3), 284 10.1039/b312471a. 14747853

[ref29] Kumar S.; Engman L. Microwave-Assisted Copper-Catalyzed Preparation of Diaryl Chalcogenides. J. Org. Chem. 2006, 71 (14), 5400–5403. 10.1021/jo060690a. 16808537

[ref30] Perin G.; Mendes S. R.; Silva M. S.; Lenardão E. J.; Jacob R. G.; Santos P. C. d. Synthesis of Β-Phenylchalcogeno-α,Β-unsaturated Ketones via Hydrochalcogenation of Acetylenes Using Microwave and Solvent-Free Conditions. Synth. Commun. 2006, 36 (18), 2587–2595. 10.1080/00397910600764162.

[ref31] Singh F. V.; Wirth T. Selenium-Catalyzed Regioselective Cyclization of Unsaturated Carboxylic Acids Using Hypervalent Iodine Oxidants. Org. Lett. 2011, 13 (24), 6504–6507. 10.1021/ol202800k. 22085140

[ref32] Cheng T.; Zheng X.; Ke Q. Ultrasound Assisted Ring-Opening Reaction of Epoxides with 1,2-DiphenylDiselenide. J. Chem. Res. 2011, 35 (9), 522–524. 10.3184/174751911X13149579868111.

[ref33] Gautheron B.; Tainturier G.; Degrand C. Ultrasound-Induced Electrochemical Synthesis of the Anions Selenide (Se22-, Se2-), and Telluride (Te22-, and Te2-). J. Am. Chem. Soc. 1985, 107 (19), 5579–5581. 10.1021/ja00305a070.

[ref34] Vukićevic R.; Konstantinovic S.; Mihailovic M. L. Electrochemical Cyclization of Unsaturated Hydroxy Compounds. Phenylselenoetherification and Phenylselenolactonization. Tetrahedron 1991, 47 (4–5), 859–865. 10.1016/S0040-4020(01)87074-5.

[ref35] García-Marín H.; van der Toorn J. C.; Mayoral J. A.; García J. I.; Arends I. W. C. E. Glycerol-Based Solvents as Green Reaction Media in Epoxidations with Hydrogen Peroxide Catalysed by Bis[3,5-Bis(Trifluoromethyl)-Diphenyl] Diselenide. Green Chem. 2009, 11 (10), 1605 10.1039/b913052g.

[ref36] Mercier E. A.; Smith C. D.; Parvez M.; Back T. G. Cyclic Seleninate Esters as Catalysts for the Oxidation of Sulfides to Sulfoxides, Epoxidation of Alkenes, and Conversion of Enamines to α-Hydroxyketones. J. Org. Chem. 2012, 77 (7), 3508–3517. 10.1021/jo300313v. 22432805

[ref37] Yu L.; Li H.; Zhang X.; Ye J.; Liu J.; Xu Q.; Lautens M. Organoselenium-Catalyzed Mild Dehydration of Aldoximes: An Unexpected Practical Method for Organonitrile Synthesis. Org. Lett. 2014, 16 (5), 1346–1349. 10.1021/ol500075h. 24564392

[ref38] Tidei C.; Piroddi M.; Galli F.; Santi C. Oxidation of Thiols Promoted by PhSeZnCl. Tetrahedron Lett. 2012, 53 (2), 232–234. 10.1016/j.tetlet.2011.11.025.

[ref39] Orian L.; Flohé L. Selenium-Catalyzed Reduction of Hydroperoxides in Chemistry and Biology. Antioxidants 2021, 10 (10), 1560 10.3390/antiox10101560. 34679695 PMC8533274

[ref40] Flohé L.; Toppo S.; Orian L. The Glutathione Peroxidase Family: Discoveries and Mechanism. Free Radical Biol. Med. 2022, 187, 113–122. 10.1016/j.freeradbiomed.2022.05.003. 35580774

[ref41] Santi C.; Tidei C.; Scalera C.; Piroddi M.; Galli F. Selenium Containing Compounds from Poison to Drug Candidates: A Review on the GPx-like Activity. Curr. Chem. Biol. 2013, 7 (1), 25–36. 10.2174/2212796811307010003.

[ref42] Bartolini D.; Torquato P.; Piroddi M.; Galli F. Targeting Glutathione S-Transferase P and Its Interactome with Selenium Compounds in Cancer Therapy. Biochim. Biophys. Acta, Gen. Subj. 2019, 1863 (1), 130–143. 10.1016/j.bbagen.2018.09.023. 30290218

[ref43] Orian L.; Mauri P.; Roveri A.; Toppo S.; Benazzi L.; Bosello-Travain V.; De Palma A.; Maiorino M.; Miotto G.; Zaccarin M.; Polimeno A.; Flohé L.; Ursini F. Selenocysteine Oxidation in Glutathione Peroxidase Catalysis: An MS-Supported Quantum Mechanics Study. Free Radical Biol. Med. 2015, 87, 1–14. 10.1016/j.freeradbiomed.2015.06.011. 26163004

[ref44] Bortoli M.; Bruschi M.; Swart M.; Orian L. Sequential Oxidations of Phenylchalcogenides by H_2_O_2_: Insights into the Redox Behavior of Selenium via DFT Analysis Analysis. New J. Chem. 2020, 44 (17), 6724–6731. 10.1039/C9NJ06449D.

[ref45] te Velde G.; Bickelhaupt F. M.; Baerends E. J.; Fonseca Guerra C.; van Gisbergen S. J. A.; Snijders J. G.; Ziegler T. Chemistry with ADF. J. Comput. Chem. 2001, 22 (9), 931–967. 10.1002/jcc.1056.

[ref46] ADF2019, AMS2020, SCM. Theoretical Chemistry, Vrije Universiteit, Amsterdam: The Netherlands. https://www.scm.com/ (accessed 2023–07–25).

[ref47] van Lenthe E.; Baerends E. J.; Snijders J. G. Relativistic Total Energy Using Regular Approximations. J. Chem. Phys. 1994, 101 (11), 9783–9792. 10.1063/1.467943.

[ref48] Handy N. C.; Cohen A. J. Left-Right Correlation Energy. Mol. Phys. 2001, 99 (5), 403–412. 10.1080/00268970010018431.

[ref49] Lee C.; Yang W.; Parr R. G. Development of the Colle-Salvetti Correlation-Energy Formula into a Functional of the Electron Density. Phys. Rev. B 1988, 37 (2), 785–789. 10.1103/PhysRevB.37.785. 9944570

[ref50] Zaccaria F.; Wolters L. P.; Fonseca Guerra C.; Orian L. Insights on Selenium and Tellurium Diaryldichalcogenides: A Benchmark DFT Study. J. Comput. Chem. 2016, 37, 1672–1680. 10.1002/jcc.24383. 27093091

[ref51] Bortoli M.; Zaccaria F.; Dalla Tiezza M.; Bruschi M.; Fonseca Guerra C.; Bickelhaupt F. M.; Orian L. Oxidation of Organic Diselenides and Ditellurides by H_2_O_2_ for Bioinspired Catalyst Design. Phys. Chem. Chem. Phys. 2018, 20 (32), 20874–20885. 10.1039/C8CP02748J. 30066704

[ref52] Zhang Y.; Sayama M.; Luo M.; Lu Y.; Tantillo D. J. Not That DDT: A Databank of Dynamics Trajectories for Organic Reactions. J. Chem. Educ. 2022, 99 (7), 2721–2725. 10.1021/acs.jchemed.2c00443.

[ref53] Klamt A. Conductor-like Screening Model for Real Solvents: A New Approach to the Quantitative Calculation of Solvation Phenomena. J. Phys. Chem. 1995, 99 (7), 2224–2235. 10.1021/j100007a062.

[ref54] Wolters L. P.; Bickelhaupt F. M. The Activation Strain Model and Molecular Orbital Theory. Wiley Interdiscip. Rev.: Comput. Mol. Sci. 2015, 5, 324–343. 10.1002/wcms.1221. 26753009 PMC4696410

[ref55] Bickelhaupt F. M.; Houk K. N. Analyzing Reaction Rates with the Distortion/Interaction-Activation Strain Model. Angew. Chem., Int. Ed. 2017, 56 (34), 10070–10086. 10.1002/anie.201701486. PMC5601271 28447369

[ref56] Bickelhaupt F. M.; Baerends E. J. Kohn-Sham Density Functional Theory: Predicting and Understanding Chemistry. In Rev. Comput. Chem.; Lipkowitz K. B., Boyd D. B., Eds.; Wiley: Indianapolis, 2000; Vol. 15, pp 1–86. 10.1002/9780470125922.ch1.

[ref57] Vermeeren P.; van der Lubbe S. C. C.; Fonseca Guerra C.; Bickelhaupt F. M.; Hamlin T. A. Understanding Chemical Reactivity Using the Activation Strain Model. Nat. Protoc. 2020, 15 (2), 649–667. 10.1038/s41596-019-0265-0. 31925400

[ref58] Zeist W.-J.; van; Guerra C. F.; Bickelhaupt F. M. PyFrag—Streamlining Your Reaction Path Analysis. J. Comput. Chem. 2008, 29 (2), 312–315. 10.1002/jcc.20786. 17557284

[ref59] Madabeni A.; Nogara P. A.; Bortoli M.; Rocha J. B. T.; Orian L. Effect of Methylmercury Binding on the Peroxide-Reducing Potential of Cysteine and Selenocysteine. Inorg. Chem. 2021, 60 (7), 4646–4656. 10.1021/acs.inorgchem.0c03619. 33587617 PMC8763373

[ref60] Goto K.; Nagahama M.; Mizushima T.; Shimada K.; Kawashima T.; Okazaki R. The First Direct Oxidative Conversion of a Selenol to a Stable Selenenic Acid: Experimental Demonstration of Three Processes Included in the Catalytic Cycle of Glutathione Peroxidase. Org. Lett. 2001, 3 (22), 3569–3572. 10.1021/ol016682s. 11678710

[ref61] Masuda R.; Kuwano S.; Sase S.; Bortoli M.; Madabeni A.; Orian L.; Goto K. Model Study on the Catalytic Cycle of Glutathione Peroxidase Utilizing Selenocysteine-Containing Tripeptides: Elucidation of the Protective Bypass Mechanism Involving Selenocysteine Selenenic Acids. Bull. Chem. Soc. Jpn. 2022, 95 (9), 1360–1379. 10.1246/bcsj.20220156.

[ref62] Goto K.; Shimada K.; Furukawa S.; Miyasaka S.; Takahashi Y.; Kawashima T. Formation of a Stable Sulfenic Acid by Hydrolysis of a Thionitrate and a Sulfenyl Bromide. Chem. Lett. 2006, 35 (8), 862–863. 10.1246/cl.2006.862.

[ref63] Masuda R.; Kimura R.; Karasaki T.; Sase S.; Goto K. Modeling the Catalytic Cycle of Glutathione Peroxidase by Nuclear Magnetic Resonance Spectroscopic Analysis of Selenocysteine Selenenic Acids. J. Am. Chem. Soc. 2021, 143 (17), 6345–6350. 10.1021/jacs.1c02383. 33887135

[ref64] Sase S.; Kakimoto R.; Goto K. Synthesis of a Stable Selenoaldehyde by Self-Catalyzed Thermal Dehydration of a Primary-Alkyl-Substituted Selenenic Acid. Angew. Chem., Int. Ed. 2015, 54 (3), 901–904. 10.1002/anie.201409485. 25411119

[ref65] Madabeni A.; Dalla Tiezza M.; Omage F. B.; Nogara P. A.; Bortoli M.; Rocha J. B. T.; Orian L. Chalcogen–Mercury Bond Formation and Disruption in Model Rabenstein’s Reactions: A Computational Analysis. J. Comput. Chem. 2020, 41 (23), 2045–2054. 10.1002/jcc.26371. 32656797

[ref66] Bortoli M.; Wolters L. P.; Orian L.; Bickelhaupt F. M. Addition–Elimination or Nucleophilic Substitution? Understanding the Energy Profiles for the Reaction of Chalcogenolates with Dichalcogenides. J. Chem. Theory Comput. 2016, 12 (6), 2752–2761. 10.1021/acs.jctc.6b00253. 27096625

[ref67] Nogara P. A.; Oliveira C. S.; Madabeni A.; Bortoli M.; Rocha J. B. T.; Orian L. Thiol Modifier Effects of Diphenyl Diselenides: Insight from Experiment and DFT Calculations. New J. Chem. 2023, 47 (12), 5796–5803. 10.1039/D2NJ05976B.

[ref68] Madabeni A.; Orian L. The Key Role of Chalcogenurane Intermediates in the Reduction Mechanism of Sulfoxides and Selenoxides by Thiols Explored In Silico. Int. J. Mol. Sci. 2023, 24 (9), 7754 10.3390/ijms24097754. 37175462 PMC10178455

[ref69] Campeggio J.; Bortoli M.; Orian L.; Zerbetto M.; Polimeno A. Multiscale Modeling of Reaction Rates: Application to Archetypal S _N_ 2 Nucleophilic Substitutions. Phys. Chem. Chem. Phys. 2020, 22 (6), 3455–3465. 10.1039/C9CP03841H. 31984980

[ref70] Nogara P. A.; Oliveira C. S.; Pereira M. E.; Bortoli M.; Orian L.; Aschner M.; Rocha J. B. T. Therapeutic Applications of Low-Molecular-Weight Thiols and Selenocompounds. In REDOX CHEMISTRY AND BIOLOGY OF THIOLS; Alvarez B., Comini M. A., Salinas G., Trujillo M., Eds.; Elsevier: Amsterdam, 2022; pp 643–665.

[ref71] Alvarez B.; Salinas G. Basic Concepts of Thiol Chemistry and Biology. In REDOX CHEMISTRY AND BIOLOGY OF THIOLS; Alvarez B., Comini M. A., Salinas G., Trujillo M., Eds.; Elsevier: Amsterdam, 2022; pp 1–16.

