# Supplementary material for: Role of Group 12 Metals in the Reduction of H2O2 by Santi’s Reagent: A Computational Mechanistic Investigation
Source: Inorg Chem. 2023 Sep 28;62(42):17288–98. doi: 10.1021/acs.inorgchem.3c02568 (PMC10598800; doi:10.1021/acs.inorgchem.3c02568)
Supplement: Supplementary file 1 — ic3c02568_si_001.pdf [file ic3c02568_si_001.pdf]

# - Supporting Information –

Role of Group 12 Metals in the Reduction of H_2O_2 by Santi's Reagent: a computational mechanistic investigation

Davide Zeppilli [a], Andrea Madabeni [a], Luca Sancineto [b], Luana Bagnoli [b], Claudio Santi [b] and Laura Orian [a,]*

[a] Dipartimento di Scienze Chimiche, Università degli Studi di Padova, Via Marzolo 1, 35131 Padova, Italy
[b] Gruppo di Catalisi Sintesi e Chimica Organica Verde Dipartimento di Scienze Farmaceutiche, Università degli Studi di Perugia, Via del Liceo 1, 06122 Perugia, Italy
[*] Correspondence: laura.orian@unipd.it

LIST of TABLES and FIGURES:

Table S1 Gibbs free energies (kcal mol^{-1}) relative to the free reactants for the reduction of H_2O_2 by **1-MCl** in water. Level of theory: COSMO-ZORA-OLYP/TZ2P……………………………………………………………………………...S3

Figure S1 Total orbital interaction energy and its contributions (kcal mol^{-1}) according to ETS-NOCV (Extended Transition State – Natural Orbital for Chemical Valence) analysis at r.c.=0.5 and dominating contributions to the deformation density. The blue/red phases corresponds to accumulation/depletion of electron density, isosuface value=0.005. Level of theory: ZORA-OLYP/TZ2P……………………………………….….……….……….….S3

Figure S2 Energy profiles (kcal mol^{-1}) relative to the free reactants for **1-ZnBr** (blue), **1-CdBr** (red) and **1-HgBr** (green) oxidation and isomerization in (A) gas phase and in (B) water. For the isomerization, the energy of a single water molecule was added to the stationary points for consistency. Levels of theory: ZORA-OLYP/TZ2P and COSMO-ZORA-OLYP/TZ2P//ZORA-OLYP/TZ2P…………………………………………………….……………………………...S4

Table S2 Gibbs free energies (kcal mol^{-1}) relative to the free reactants for the reduction of H_2O_2 by **1-MBr** in the gas phase. Level of theory: ZORA-OLYP/TZ2P…………………...……………………………………………………...S4

Figure S3 (A) Activation strain analysis of **1-ZnBr** (blue lines), **1-CdBr** (red lines) and **1-HgBr** (green lines) oxidations: energy profiles (solid lines), strain contributions (dashed lines), interaction contributions (dash-dotted lines). Energy decomposition analysis: (B) Pauli repulsion, (C) electrostatic interaction and (D) orbital interaction. The position of the transition states is indicated by filled dots. The reaction coordinate is defined as: r.c.= (d_{O-O}-d_{O-O}^0), where d_{O-O}^0 represents the O–O bond length in the reactant complex of each reaction. Level of theory: ZORA-OLYP/TZ2P…………………...S5

Table S3 Gibbs free energies (kcal mol^{-1}) relative to the free reactants for the reduction of H_2O_2 by **1-MBr** in water. Level of theory: COSMO-ZORA-OLYP/TZ2P// ZORA-OLYP/TZ2P………………...………………………………...S5

Table S4 Electronic energies (kcal mol^{-1}) relative to the free reactants for the reduction of H_2O_2 by **1-MI** in the gas phase. Activation energies relative to reactant complexes are given in parentheses. Level of theory: ZORA-OLYP/TZ2P……..S6

Table S5 Gibbs free reaction energies (kcal mol^{-1}) for the hydration equilibria of **2-ZnX**, **2-CdX** and **4-HgX** in the gas phase and in water. Levels of theory: ZORA-OLYP/TZ2P and COSMO-ZORA-OLYP/TZ2P//ZORA-OLYP/TZ2P…..S6

Table S6 Coordinates (Å) and energies (E, Hartree) of stationary points and number of imaginary frequencies (Nimag, cm^{-1}) of transition states. Level of theory: ZORA-OLYP/TZ2P………………………………………………………….S6

Table S7 Coordinates (Å) and energies (E, Hartree) of stationary points and number of imaginary frequencies (Nimag, cm^{-1}) of transition states. Level of theory: COSMO-ZORA-OLYP/TZ2P………………………………………………S19

Table S1 Gibbs free reaction energies (kcal mol^{-1}) relative to the free reactants for the reduction of H_2O_2 by **1-MCl** in water. Level of theory: COSMO-ZORA-OLYP/TZ2P.

	**RC**	**TS**	**PC**	**2-MCl**	**4-MCl**	**5-MCl**
**Zn**	10.85	17.32	-36.56	-38.41	-30.06	-35.03
**Cd**	10.83	19.86	-29.20	-33.30	-33.65	-29.27
**Hg**	8.21	21.08	-31.86	-26.82	-34.98	-

Figure S1 Total orbital interaction energy and its contributions (kcal mol^{-1}) according to ETS-NOCV (Extended Transition State – Natural Orbital for Chemical Valence) analysis at r.c.=0.5 and dominating contributions to the deformation density. The blue/red phases corresponds to accumulation/depletion of electron density, isosuface value=0.005. Level of theory: ZORA-OLYP/TZ2P.

	**Total**	**1**	**2**	**3**	**4**	**5**	**6**	**7**
**Zn**	-87.41	-63.97	-15.12	-2.62	-1.83	-1.89	-0.57	-0.12
**Cd**	-86.06	-67.87	-9.56	-2.80	-1.88	-1.49	-1.02	-0.17
**Hg**	-72.16	-59.85	-5.42	-3.76	-1.74	-0.45	-0.22	-0.14

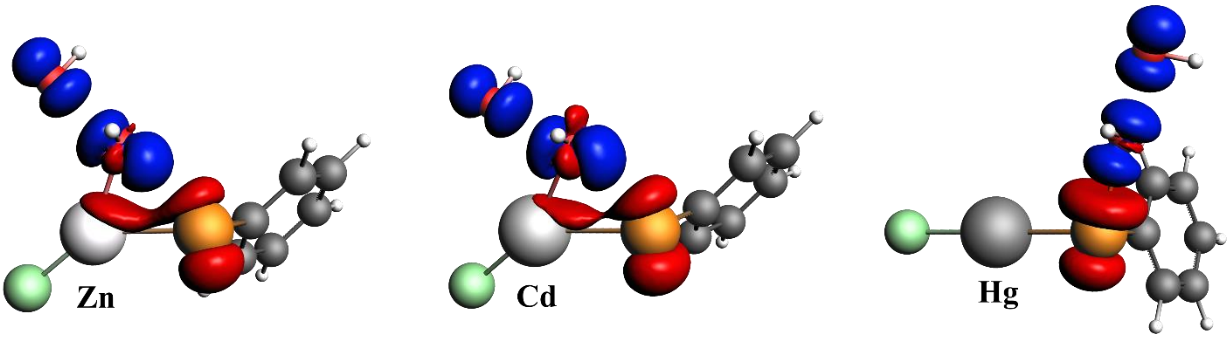

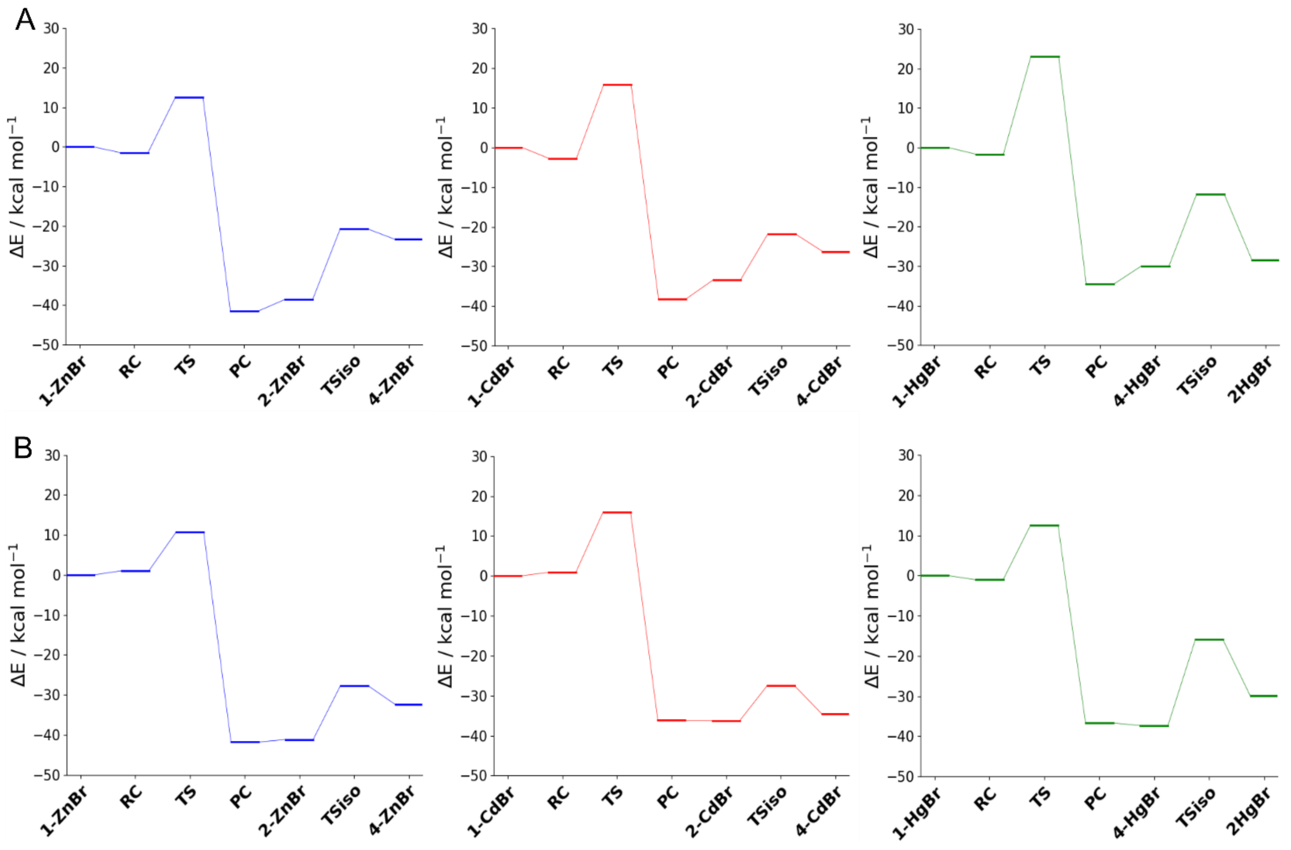

Figure S2 Energy profiles (kcal mol^{-1}) relative to the free reactants for **1-ZnBr** (blue), **1-CdBr** (red) and **1-HgBr** (green) oxidation and isomerization in (A) gas phase and in (B) water. For the isomerization, the energy of a single water molecule was added to the stationary points for consistency. Levels of theory: ZORA-OLYP/TZ2P and COSMO-ZORA-OLYP/TZ2P//ZORA-OLYP/TZ2P

Table S2 Gibbs free reaction energies (kcal mol^{-1}) relative to the free reactants for the reduction of H_2O_2 by **1-MBr** in the gas phase. Level of theory: ZORA-OLYP/TZ2P.

	**RC**	**TS**	**PC**	**2-MBr**	**4-MBr**	**5-MBr**
**Zn**	9.01	20.00	-31.95	-38.67	-24.97	-36.47
**Cd**	7.06	23.75	-27.77	-33.54	-27.80	-28.87
**Hg**	7.40	31.04	-24.86	-28.57	-31.45	-19.15

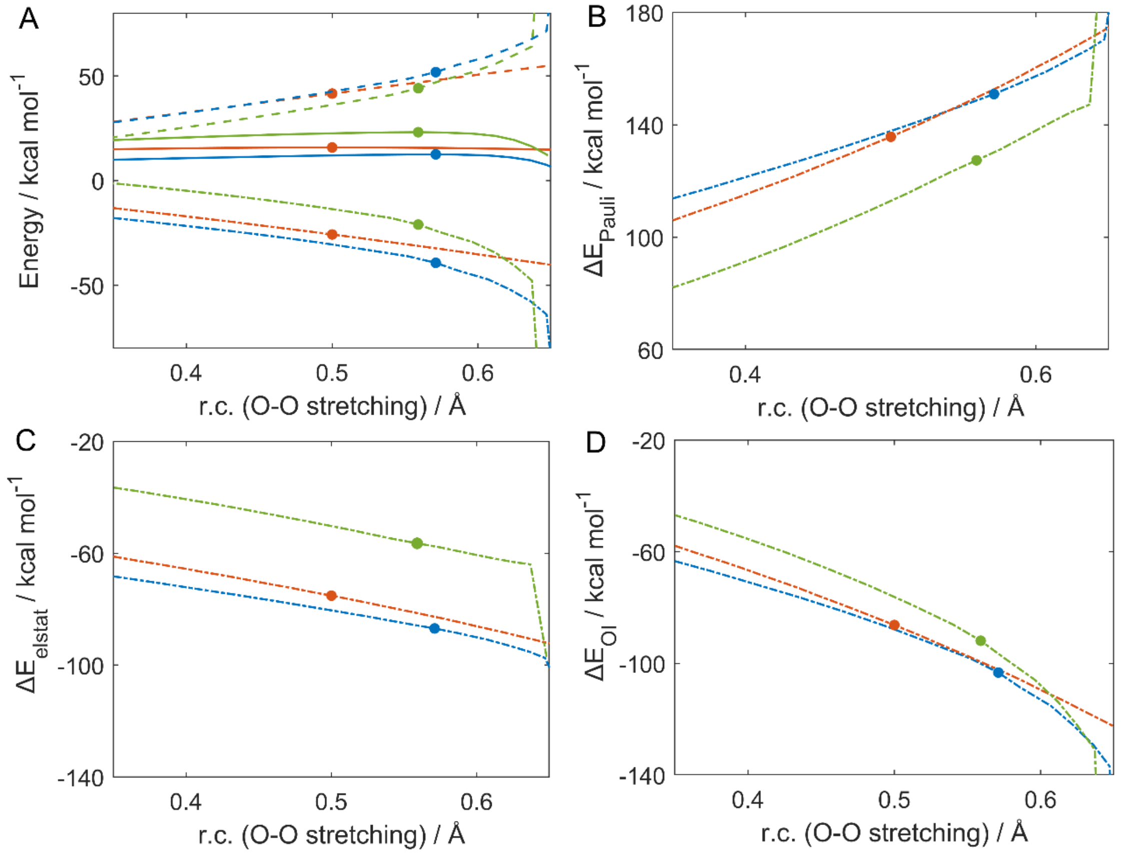

Figure S3 (A) Activation strain analysis of **1-ZnBr** (blue lines), **1-CdBr** (red lines) and **1-HgBr** (green lines) oxidations: energy profiles (solid lines), strain contributions (dashed lines), interaction contributions (dash-dotted lines). Energy decomposition analysis: (B) Pauli repulsion, (C) electrostatic interaction and (D) orbital interaction. The position of the transition states is indicated by filled dots. The reaction coordinate is defined as: r.c.= ($d_{O\text{-}O}$-$d_{O\text{-}O}^0$), where $d_{O\text{-}O}^0$ represents the O–O bond length in the reactant complex of each reaction. Level of theory: ZORA-OLYP/TZ2P.

Table S3 Gibbs free reaction energies (kcal mol^{-1}) relative to the free reactants for the reduction of H_2O_2 by **1-MBr** in water. Level of theory: COSMO-ZORA-OLYP/TZ2P// ZORA-OLYP/TZ2P.

	**RC**	**TS**	**PC**	**2-MBr**	**4-MBr**	**5-MBr**
**Zn**	9.62	16.33	-34.11	-38.96	-31.64	-36.23
**Cd**	8.84	22.05	-26.38	-34.00	-33.78	-29.10
**Hg**	6.14	18.61	-28.87	-27.77	-36.44	-19.99

Table S4 Electronic energies (kcal mol^{-1}) relative to the free reactants for the reduction of H_2O_2 by **1-MI** in the gas phase. Activation energies relative to reactant complexes are given in parentheses. Level of theory: ZORA-OLYP/TZ2P.

	**RC**	**TS**	**PC**	**2-MI**	**4-MI**
**Zn**	-1.28	12.21 (13.49)	-41.86	-38.79	-23.66
**Cd**	-1.83	15.30 (17.13)	-38.56	-33.81	-26.42
**Hg**	-2.08	22.73 (24.81)	-34.87	-28.96	-30.25

Table S5 Gibbs free reaction energies (kcal mol^{-1}) for the hydration equilibria of **2-ZnX**, **2-CdX** and **4-HgX** in the gas phase and in water. Levels of theory: ZORA-OLYP/TZ2P and COSMO-ZORA-OLYP/TZ2P//ZORA-OLYP/TZ2P.

	$\Delta G_{gas\ phase}$			ΔG_{water}		
	**Zn**	**Cd**	**Hg**	**Zn**	**Cd**	**Hg**
**Cl**	-1.86	-0.51	2.59	-2.07	-0.92	8.33
**Br**	-1.84	-0.45	2.92	-1.99	-0.86	8.25

Table S6 Coordinates (Å) and energies (E, Hartree) of stationary points and number of imaginary frequencies (Nimag, cm^{-1}) of transition states. Level of theory: ZORA-OLYP/TZ2P.

H_2O_2 E= -0.64253371

O	-0.406570	-0.627582	0.024045
O	0.406570	0.627582	0.024045
H	-0.179185	1.198153	-0.513228
H	0.179185	-1.198153	-0.513228

H_2O E= -0.50675052

O	0.000000	0.000000	-0.000193
H	0.000000	-0.768778	-0.595946
H	0.000000	0.768778	-0.595946

1-ZnCl E= -2.80363438

Se	0.804589	-1.769833	-0.066696
Zn	-0.018170	0.280860	-0.652602
Cl	-0.769871	2.178274	-1.192944
C	-3.234437	-4.237996	-0.432709
C	-2.886907	-3.637920	0.777162
C	-2.403286	-4.086656	-1.542212
H	-3.526424	-3.753763	1.648895
H	-2.663801	-4.554259	-2.488690
C	-1.715879	-2.886039	0.880737
C	-1.230189	-3.336809	-1.447644
H	-1.447162	-2.425558	1.826469
H	-0.583693	-3.227362	-2.312933
C	-0.887737	-2.729846	-0.235217
H	-4.147378	-4.822712	-0.509948

RC E= -3.45913989

Se	0.747567	-3.384696	-0.143298
Zn	0.112098	-1.561246	-1.388254
Cl	-0.801990	0.066735	-2.398330
C	-3.528402	-4.905696	1.246974
C	-2.809571	-4.009627	2.037528
C	-2.995495	-5.333510	0.031325
H	-3.215037	-3.672062	2.988409
H	-3.546952	-6.034319	-0.591010
C	-1.565199	-3.538789	1.616657
C	-1.751178	-4.868412	-0.395978
H	-1.008825	-2.843354	2.237602
H	-1.339092	-5.209642	-1.340720
C	-1.034740	-3.963979	0.394428
H	-4.497898	-5.269757	1.577101
H	3.230229	-2.123141	-2.535458
O	3.371172	-0.248153	-2.315038

H	3.319877	0.233259	-3.157626
O	2.569059	-1.421256	-2.652436

TS E= -3.43620774

Nimag= -469.252

Se	1.232266	-3.299629	0.547228
Zn	1.039654	-0.892766	-0.094423
Cl	0.692454	1.061076	0.625276
C	-3.242069	-4.762056	0.495569
C	-2.919295	-3.566149	1.138252
C	-2.243030	-5.502409	-0.138838
H	-3.692236	-2.987238	1.637214
H	-2.489100	-6.435194	-0.639928
C	-1.605091	-3.104396	1.146105
C	-0.925412	-5.052143	-0.135481
H	-1.361810	-2.179074	1.658862
H	-0.151114	-5.628764	-0.632173
C	-0.601309	-3.846628	0.505522
H	-4.269349	-5.116777	0.490094
H	2.195630	-2.199798	-1.924895
O	1.810452	-1.164124	-3.310958
H	1.185930	-1.674062	-3.850591
O	1.278289	-2.113484	-1.596155

PC E= -3.52628638

Se	0.888541	-2.868292	1.091475
Zn	0.986380	-0.849993	-1.290021
Cl	0.243540	1.072941	-1.749499
C	-3.341333	-4.909451	0.583244
C	-2.931579	-4.391454	1.813285
C	-2.494006	-4.805296	-0.519864
H	-3.586163	-4.457791	2.679259
H	-2.802352	-5.207159	-1.482606
C	-1.693004	-3.765893	1.938995
C	-1.242286	-4.201110	-0.400992
H	-1.401163	-3.341230	2.897113
H	-0.578987	-4.142821	-1.257970
C	-0.839937	-3.675644	0.830599
H	-4.313062	-5.386056	0.485859
H	2.588729	-2.311501	-3.074923
O	2.420446	-1.359185	-3.075331
H	2.176110	-1.124478	-3.980087
O	1.385947	-2.502051	-0.612629

2-ZnCl E= -3.00276674

Se	0.583419	-1.715004	0.548757
Zn	0.022167	0.795071	-1.053163
Cl	-0.897842	2.662666	-1.198171
C	-3.195426	-4.349811	-0.484456
C	-2.907677	-3.992634	0.833337
C	-2.368683	-3.901570	-1.515488
H	-3.550440	-4.328516	1.643599
H	-2.584367	-4.176917	-2.545319
C	-1.810696	-3.181442	1.120502
C	-1.251330	-3.114730	-1.238310
H	-1.617034	-2.881514	2.147768
H	-0.597752	-2.789230	-2.041458
C	-0.974894	-2.748908	0.083016
H	-4.059835	-4.969887	-0.706462
O	0.875742	-0.791827	-1.001943

TS_{iso} E= -2.97412327

Nimag= -162.439

Se	0.623572	-1.495357	0.276007
Zn	-0.200452	0.577064	-0.402967
Cl	-0.774107	2.392489	-1.304639
C	-3.160366	-4.270167	-0.472636
C	-2.894026	-3.798656	0.814578
C	-2.330901	-3.908699	-1.534291
H	-3.535519	-4.083471	1.644930
H	-2.532776	-4.280845	-2.535872
C	-1.799530	-2.965049	1.043472
C	-1.236432	-3.068480	-1.320105
H	-1.590964	-2.609177	2.050449
H	-0.575298	-2.779816	-2.133142
C	-0.989591	-2.601849	-0.032866
H	-4.012210	-4.922630	-0.645408
O	1.334534	-1.292913	-1.267844

4-ZnCl E= -2.97828963

Se	0.897178	-1.864492	0.034782
Zn	-0.076778	0.310547	-0.465770
Cl	-0.848357	2.212493	-1.010240
C	-3.226261	-4.201475	-0.455965
C	-2.946823	-3.544905	0.744164
C	-2.312155	-4.151302	-1.508568
H	-3.652192	-3.591161	1.570243
H	-2.524433	-4.669877	-2.440490
C	-1.756358	-2.832680	0.892322
C	-1.118563	-3.439411	-1.373021
H	-1.539343	-2.334283	1.835364
H	-0.387601	-3.400726	-2.176514
C	-0.861616	-2.778529	-0.176498
H	-4.153824	-4.757035	-0.566569
O	1.751461	-2.225500	-1.349364

5-ZnCl E= -3.53039726

Se	-0.570837	-1.571703	-0.748554
Cl	1.582686	-1.426415	-4.303417
C	-2.441506	-5.716505	-2.043912
C	-1.698140	-5.639621	-0.866946
C	-2.611987	-4.576749	-2.830454

H	-1.563557	-6.522860	-0.247218
H	-3.188155	-4.626968	-3.750860
C	-1.121155	-4.432486	-0.468401
C	-2.042343	-3.363652	-2.446187
H	-0.548553	-4.370770	0.450946
H	-2.178599	-2.484662	-3.071774
C	-1.296638	-3.304010	-1.266379
H	-2.887198	-6.659806	-2.347813
Zn	1.735254	-1.735077	-2.206907
H	1.263528	-2.102849	0.431802
O	2.622979	-2.019594	-0.613733
H	3.343661	-2.657238	-0.657631
O	0.323342	-1.979383	0.750452

1-CdCl E= -2.78392918

Se	0.749041	-1.979784	0.294449
Cl	-1.430040	2.277766	0.151966
C	-3.041329	-4.830050	0.476491
C	-2.461508	-4.358037	1.653779
C	-2.518614	-4.437382	-0.755439
H	-2.858555	-4.662607	2.619189
H	-2.960385	-4.804296	-1.678837
C	-1.366849	-3.494186	1.603627
C	-1.423839	-3.574233	-0.814218
H	-0.915647	-3.135046	2.523575
H	-1.016613	-3.278097	-1.776177
C	-0.847734	-3.094605	0.367271
H	-3.894090	-5.502625	0.518787
Cd	-0.382378	0.217403	0.221666

RC E= -3.44230249

Se	-0.358412	-2.800876	0.063467
Cl	3.335785	-2.565636	-3.014304
C	-3.135778	-5.686349	-2.481689
C	-2.273645	-6.209907	-1.518704
C	-3.155930	-4.311291	-2.714360
H	-2.253999	-7.280012	-1.326382
H	-3.828340	-3.892645	-3.459411
C	-1.430925	-5.366660	-0.793405
C	-2.318706	-3.461354	-1.990978
H	-0.764665	-5.779782	-0.042167
H	-2.347006	-2.390687	-2.169442
C	-1.447087	-3.987978	-1.031222
H	-3.790396	-6.345680	-3.045725
Cd	1.598536	-2.598384	-1.440866
H	3.957259	-1.504541	0.888956
O	4.524295	-0.499595	-0.611605
H	4.429147	-1.056354	-1.410748
O	3.444027	-1.036478	0.209934

TS E= -3.41095618

Nimag= -179.053

Se	-0.681094	-1.693619	-0.499654
Cl	1.820249	-0.271932	-4.378436
C	-2.416031	-5.849443	-1.893231
C	-2.115388	-5.615126	-0.550364
C	-2.186254	-4.849455	-2.838379
H	-2.293774	-6.388848	0.192297
H	-2.421860	-5.021743	-3.885593
C	-1.587311	-4.390266	-0.150186
C	-1.652902	-3.622021	-2.449314
H	-1.356530	-4.213652	0.895951
H	-1.491629	-2.847180	-3.192658
C	-1.347739	-3.386127	-1.100546
H	-2.829231	-6.806302	-2.201072
Cd	1.319879	-1.174138	-2.306951
H	1.954209	-1.701055	0.312461
O	3.608081	-2.275376	-0.899598
H	3.677093	-3.241661	-0.858846
O	1.704055	-2.262239	-0.439607

5-CdCl E= -3.00053362

Se	-0.636029	-1.567293	-0.707169
Cl	1.673314	-1.244593	-4.616727
C	-2.437507	-5.731433	-2.040141
C	-1.689097	-5.652975	-0.866299
C	-2.631665	-4.586267	-2.813574
H	-1.536572	-6.539896	-0.255900
H	-3.211877	-4.636781	-3.731516
C	-1.132062	-4.439882	-0.457858
C	-2.080212	-3.367967	-2.419650
H	-0.557009	-4.376784	0.460049
H	-2.235531	-2.485110	-3.035856
C	-1.329814	-3.304490	-1.242013
H	-2.868526	-6.679110	-2.351445
Cd	1.876775	-1.674224	-2.350992
H	1.264922	-2.082685	0.404037
O	2.682770	-2.028117	-0.486946
H	3.247106	-2.810630	-0.472434
O	0.323794	-1.982112	0.747445

TS2 E= -3.49466491

Nimag= -293.527

Se	-0.577455	-1.601454	-0.706679
Cl	2.430490	-1.422296	-4.513627
C	-2.485280	-5.738168	-2.024684
C	-1.627117	-5.686445	-0.926803
C	-2.764966	-4.574278	-2.742983
H	-1.412638	-6.588603	-0.358868
H	-3.436384	-4.606167	-3.597408
C	-1.039318	-4.479347	-0.543326
C	-2.187673	-3.361590	-2.368644

H	-0.378947	-4.426504	0.316956
H	-2.417351	-2.459688	-2.932351
C	-1.320777	-3.328351	-1.274405
H	-2.939128	-6.680756	-2.318648
Cd	1.580860	-1.614160	-2.350739
H	1.599257	-2.052104	0.206750
O	2.599316	-1.976878	-0.388315
H	3.076713	-2.810630	-0.303421
O	0.411944	-2.010317	0.660377

PC E= -3.49586400

Se	-0.564749	-1.622009	-0.675374
Cl	2.677496	-1.518080	-4.389236
C	-2.501929	-5.745190	-2.027430
C	-1.629080	-5.703055	-0.940642
C	-2.791624	-4.575657	-2.733125
H	-1.408775	-6.609967	-0.382496
H	-3.476872	-4.601165	-3.576799
C	-1.035066	-4.498821	-0.557151
C	-2.207555	-3.366438	-2.357660
H	-0.363983	-4.448461	0.295577
H	-2.446377	-2.458969	-2.908849
C	-1.323812	-3.343590	-1.277355
H	-2.960979	-6.685107	-2.322110
Cd	1.435462	-1.587693	-2.414691
H	1.868613	-2.024226	0.194303
O	2.705317	-1.910843	-0.385762
H	3.227874	-2.715517	-0.285574
O	0.372584	-2.025950	0.689556

4-CdCl E= -2.96328529

Se	-0.652500	-1.540867	-0.731073
Cl	2.876525	-1.436127	-4.224227
C	-2.468931	-5.753653	-2.004786
C	-1.538611	-5.670290	-0.968514
C	-2.855713	-4.600570	-2.690740
H	-1.243736	-6.566009	-0.427039
H	-3.588821	-4.660282	-3.491411
C	-0.983016	-4.438675	-0.616650
C	-2.308811	-3.364400	-2.346377
H	-0.268020	-4.352223	0.197369
H	-2.624520	-2.468156	-2.877540
C	-1.366722	-3.300833	-1.319109
H	-2.898648	-6.714760	-2.274625
Cd	1.176455	-1.477725	-2.627865
O	0.218672	-1.865281	0.649544

TS_{iso} E= -2.95599196

Nimag= -149.389

Se	-0.610112	-1.463358	-1.249382
Cl	3.473155	-1.499880	-3.790068
C	-2.510649	-5.760056	-1.909085
C	-1.359435	-5.613323	-1.134865
C	-3.095701	-4.642373	-2.508179
H	-0.906988	-6.480928	-0.660445
H	-3.997123	-4.751686	-3.106248
C	-0.780146	-4.354954	-0.962191
C	-2.531708	-3.378673	-2.336957
H	0.112419	-4.221443	-0.356637
H	-2.999244	-2.510513	-2.797200
C	-1.370234	-3.251210	-1.572597
H	-2.955630	-6.742568	-2.042672
Cd	1.436155	-1.449568	-2.709998
O	0.780340	-1.741311	-0.256931

2-CdCl E= -2.97458599

Se	-0.929247	-1.340102	-1.165812
Cl	3.645133	-1.646472	-4.020882
C	-2.447374	-5.738535	-1.977277
C	-1.286111	-5.539377	-1.230188
C	-3.133772	-4.635727	-2.488625
H	-0.747110	-6.392210	-0.823722
H	-4.035956	-4.779335	-3.078581
C	-0.815078	-4.250554	-0.979764
C	-2.657584	-3.344578	-2.268919
H	0.071638	-4.099361	-0.372070
H	-3.184556	-2.496274	-2.700051
C	-1.498638	-3.149154	-1.505486
H	-2.814067	-6.744695	-2.161867
Cd	2.111462	-1.621658	-2.320959
O	0.820502	-1.573943	-0.765070

1-HgCl E= -2.74900459

Se	0.717621	-1.961644	0.319941
Cl	-1.375874	2.332634	0.089765
C	-3.034215	-4.850085	0.475759
C	-2.455349	-4.388273	1.657600
C	-2.523526	-4.434514	-0.753915
H	-2.845173	-4.710776	2.620010
H	-2.966851	-4.793445	-1.679585
C	-1.371985	-3.510203	1.614505
C	-1.439023	-3.558586	-0.806095
H	-0.922132	-3.154622	2.536374
H	-1.039921	-3.242874	-1.765113
C	-0.864549	-3.090019	0.380414
H	-3.878942	-5.533103	0.512680
Hg	-0.368619	0.239735	0.203790

RC E= -3.40496299

Se	0.921016	-2.656042	0.704739
Cl	-0.108696	1.879647	-0.419276
C	-3.342595	-4.706670	0.482567

C	-2.883110	-4.165310	1.682939
C	-2.554818	-4.616768	-0.664702
H	-3.489895	-4.234159	2.582462
H	-2.904631	-5.039300	-1.603337
C	-1.641046	-3.532754	1.739106
C	-1.310810	-3.986621	-0.619385
H	-1.284540	-3.115704	2.675960
H	-0.703235	-3.925043	-1.516259
C	-0.856027	-3.440125	0.585058
H	-4.310915	-5.198692	0.441754
H	1.950090	-3.191167	-1.808132
O	1.984966	-2.110099	-3.364954
H	2.904384	-1.898106	-3.593643
O	2.174434	-3.413833	-2.731680
Hg	0.379211	-0.328389	0.105213

TS E= -3.36453971

Nimag= -530.751

Se	0.730049	-1.985195	0.408396
Cl	-1.437935	2.298161	-0.052387
C	-3.042340	-4.818430	0.470781
C	-2.443988	-4.409832	1.662064
C	-2.533271	-4.379378	-0.751839
H	-2.829909	-4.755455	2.617542
H	-2.991186	-4.703565	-1.682822
C	-1.340079	-3.557246	1.635610
C	-1.430551	-3.526732	-0.796268
H	-0.871381	-3.244918	2.564238
H	-1.019786	-3.203878	-1.746244
C	-0.847444	-3.117538	0.404350
H	-3.901881	-5.483128	0.494620
H	2.300153	-2.298300	-1.611176
O	2.487474	-3.131815	-3.069965
H	2.547969	-4.062969	-2.807945
O	1.376755	-2.607845	-1.468235
Hg	-0.408837	0.231357	0.178089

PC E= -3.45676673

Se	1.043949	-2.643951	0.605818
Cl	-0.373368	1.739712	-1.180938
C	-3.160410	-4.846035	0.462949
C	-2.876952	-3.954596	1.499171
C	-2.220153	-5.071185	-0.542655
H	-3.604525	-3.782939	2.288404
H	-2.437653	-5.769524	-1.347030
C	-1.654712	-3.283464	1.529875
C	-0.994534	-4.403539	-0.524905
H	-1.436151	-2.596618	2.345157
H	-0.250108	-4.576649	-1.296264
C	-0.732271	-3.509983	0.508194
H	-4.113342	-5.368225	0.442976
H	1.718826	-2.483506	-2.275422
O	1.355436	-1.835424	-2.911735
H	2.127039	-1.460222	-3.352380
O	1.944197	-3.368024	-0.598690
Hg	0.285152	-0.381376	-0.414266

4-HgCl E= -2.93415965

Se	1.059162	-2.659546	0.614471
Cl	-0.381630	1.703167	-1.273336
C	-3.160367	-4.838804	0.457448
C	-2.873946	-3.963694	1.506808
C	-2.221964	-5.051277	-0.553104
H	-3.600127	-3.803659	2.299803
H	-2.441985	-5.738698	-1.366250
C	-1.650581	-3.294567	1.545294
C	-0.994999	-4.386522	-0.526100
H	-1.429269	-2.620341	2.370453
H	-0.246345	-4.549687	-1.296590
C	-0.730208	-3.507962	0.519234
H	-4.114124	-5.359296	0.431541
O	1.934688	-3.373583	-0.594764
Hg	0.270814	-0.371925	-0.357113

TS_{iso} E= -2.90412343

Nimag= -184.193

Se	0.694567	-2.184240	0.900466
Cl	0.177863	1.646272	-2.057081
C	-3.106311	-4.946213	0.426391
C	-2.888594	-4.247479	1.616030
C	-2.207438	-4.814242	-0.632326
H	-3.583524	-4.350562	2.445781
H	-2.370386	-5.361925	-1.557483
C	-1.776240	-3.419072	1.750278
C	-1.094741	-3.980256	-0.516397
H	-1.607851	-2.886584	2.683966
H	-0.384437	-3.871885	-1.330799
C	-0.890922	-3.286865	0.675947
H	-3.973403	-5.593867	0.327761
O	1.541282	-2.332358	-0.671883
Hg	0.520184	-0.234329	-0.772097

2-HgCl E= -2.93164994

Se	0.930254	-2.511948	1.009010
Cl	-0.453203	1.869287	-1.919278
C	-3.145661	-4.819464	0.424131
C	-2.846133	-4.194113	1.634585
C	-2.240497	-4.743340	-0.636198
H	-3.549873	-4.239563	2.462262
H	-2.467083	-5.230680	-1.581646
C	-1.656078	-3.482194	1.782903
C	-1.033087	-4.063353	-0.488189

H	-1.448108	-2.965992	2.716998
H	-0.319907	-4.028818	-1.305956
C	-0.742092	-3.425030	0.722890
H	-4.080893	-5.360283	0.305824
O	1.291559	-1.880356	-0.649574
Hg	0.448133	-0.117943	-1.210876

5-HgCl E= -3.45004884

Se	-0.864493	-1.559882	-0.577355
Cl	1.991972	-1.058100	-4.722494
C	-2.430956	-5.770532	-2.042271
C	-1.703935	-5.686622	-0.854924
C	-2.668217	-4.613106	-2.785264
H	-1.516519	-6.581940	-0.266390
H	-3.230227	-4.665056	-3.714739
C	-1.217966	-4.459508	-0.402060
C	-2.178967	-3.382672	-2.350153
H	-0.664270	-4.396826	0.529018
H	-2.361152	-2.492492	-2.948817
C	-1.454763	-3.307287	-1.154404
H	-2.809598	-6.729015	-2.386930
Hg	2.383492	-1.610631	-2.549933
H	1.128840	-2.050522	0.394406
O	2.775428	-2.015726	-0.614783
H	3.307309	-2.823748	-0.586160
O	0.236802	-1.986682	0.796265

1-ZnBr E= -2.78500571

Se	1.073884	-3.051767	1.349469
Zn	0.966413	-1.171477	0.043210
C	-3.255994	-4.758422	0.411995
C	-3.067001	-3.870993	1.470988
C	-2.171779	-5.121115	-0.386526
H	-3.905498	-3.585492	2.101686
H	-2.308463	-5.815746	-1.211858
C	-1.801695	-3.343502	1.731884
C	-0.903039	-4.598342	-0.132932
H	-1.659097	-2.657525	2.561201
H	-0.061351	-4.888483	-0.754249
C	-0.717234	-3.702922	0.925197
H	-4.242940	-5.167172	0.211458
Br	0.867400	0.678721	-1.237944

RC E= -3.44018760

Se	0.763487	-3.389821	-0.036225
Zn	0.166318	-1.557478	-1.297811
C	-3.552622	-4.887081	1.251328
C	-2.845307	-3.999672	2.061830
C	-2.995986	-5.312676	0.045553
H	-3.269401	-3.663775	3.005159
H	-3.538418	-6.006597	-0.592256
C	-1.588815	-3.535420	1.670725
C	-1.739393	-4.854221	-0.351911
H	-1.041731	-2.846595	2.307098
H	-1.308955	-5.193827	-1.289021
C	-1.033972	-3.958769	0.458585
H	-4.531556	-5.246122	1.558328
H	3.323068	-2.230049	-2.369473
O	3.540405	-0.359735	-2.172534
H	3.548040	0.102548	-3.027185
O	2.697469	-1.501756	-2.516848
Br	-0.775710	0.185988	-2.394109

TS E= -3.41785873

Nimag= -465.832

Se	1.215403	-3.215067	0.552539
Zn	0.984356	-0.821562	-0.140204
C	-3.228053	-4.769512	0.499131
C	-2.934151	-3.554816	1.120007
C	-2.209638	-5.501567	-0.113706
H	-3.722275	-2.981964	1.601964
H	-2.433050	-6.448901	-0.597843
C	-1.629659	-3.066204	1.127590
C	-0.901484	-5.024406	-0.110340
H	-1.408924	-2.125894	1.622826
H	-0.112072	-5.594778	-0.590179
C	-0.606441	-3.800220	0.508827
H	-4.247848	-5.145232	0.493790
H	2.172955	-2.159407	-1.935117
O	1.773978	-1.164845	-3.349787
H	1.160369	-1.700108	-3.877221
O	1.252863	-2.078977	-1.613029
Br	0.576992	1.276000	0.567983

PC E= -3.50405912

Se	1.085070	-2.617792	0.837907
Zn	0.420109	-0.016950	-0.592006
C	-3.071293	-4.825276	0.485877
C	-2.650567	-4.283097	1.699658
C	-2.269238	-4.689183	-0.649192
H	-3.273136	-4.376227	2.586201
H	-2.591329	-5.111271	-1.598155
C	-1.441674	-3.590942	1.777846
C	-1.041871	-4.033679	-0.574872
H	-1.137381	-3.136731	2.717319
H	-0.414077	-3.957988	-1.456645
C	-0.630589	-3.475768	0.641929
H	-4.021709	-5.348707	0.422348
H	1.674272	-2.524667	-2.573631
O	1.689662	-2.832897	-3.492815
H	2.577537	-3.191376	-3.608844
O	1.193714	-1.649439	-0.720136

Br	-0.465177	2.020530	-0.475555

2-ZnBr E= -2.98430486

Se	0.544212	-1.649807	0.566296
Zn	-0.030873	0.875981	-1.021376
C	-3.184210	-4.348347	-0.487429
C	-2.890753	-4.012767	0.834883
C	-2.378415	-3.859280	-1.516366
H	-3.517076	-4.380689	1.644144
H	-2.598300	-4.117384	-2.549794
C	-1.809264	-3.183517	1.128689
C	-1.276601	-3.052677	-1.233357
H	-1.611465	-2.902446	2.160556
H	-0.639090	-2.694947	-2.035660
C	-0.994441	-2.709228	0.092632
H	-4.036561	-4.983228	-0.714180
O	0.825059	-0.715795	-0.977702
Br	-1.008936	2.871550	-1.164750

TS_{iso} E= -2.95594821

Nimag= -162.46

Se	0.628920	-1.513639	0.289422
Zn	-0.189728	0.569332	-0.392190
Br	-0.795145	2.507833	-1.356554
C	-3.163338	-4.272362	-0.475717
C	-2.898697	-3.804523	0.813215
C	-2.329031	-3.912406	-1.534115
H	-3.543954	-4.088112	1.641089
H	-2.529550	-4.281678	-2.537051
C	-1.801345	-2.976073	1.046940
C	-1.231611	-3.077397	-1.314943
H	-1.594394	-2.622918	2.055221
H	-0.566809	-2.789864	-2.125386
C	-0.986275	-2.614138	-0.026085
H	-4.017574	-4.920666	-0.652351
O	1.344464	-1.310947	-1.251828

4-ZnBr E= -2.96014410

Se	0.874556	-1.803129	0.017301
Zn	-0.127321	0.367345	-0.497030
C	-3.218641	-4.195089	-0.453577
C	-2.946917	-3.525706	0.741256
C	-2.305742	-4.141373	-1.507082
H	-3.651291	-3.574400	1.568058
H	-2.511941	-4.669711	-2.434890
C	-1.765483	-2.797419	0.883148
C	-1.121184	-3.413514	-1.377692
H	-1.554354	-2.288951	1.822141
H	-0.391168	-3.371719	-2.181881
C	-0.871617	-2.740341	-0.186358
H	-4.139135	-4.763218	-0.559352
O	1.732356	-2.163669	-1.364935
Br	-0.970030	2.387266	-1.089002

5-ZnBr E= -3.51181076

Se	-0.571842	-1.573909	-0.747563
Br	1.644236	-1.403775	-4.429157
C	-2.444181	-5.718312	-2.041724
C	-1.691158	-5.643864	-0.870750
C	-2.624622	-4.575992	-2.822291
H	-1.549042	-6.529067	-0.255529
H	-3.208816	-4.624221	-3.737735
C	-1.113724	-4.436783	-0.472718
C	-2.055519	-3.362715	-2.437863
H	-0.533453	-4.376960	0.441920
H	-2.200618	-2.481360	-3.058054
C	-1.299367	-3.305718	-1.264641
H	-2.889902	-6.661659	-2.345440
Zn	1.739094	-1.732607	-2.188389
H	1.256329	-2.102996	0.442824
O	2.616471	-2.020633	-0.584253
H	3.335992	-2.660001	-0.622120
O	0.312903	-1.979777	0.756498

1-CdBr E= -2.76798193

Se	0.708032	-1.899059	0.290254
C	-3.028731	-4.819057	0.475517
C	-2.455824	-4.337897	1.652525
C	-2.514503	-4.415964	-0.756622
H	-2.846113	-4.650456	2.618156
H	-2.950808	-4.789806	-1.679861
C	-1.376707	-3.454794	1.601837
C	-1.435589	-3.533100	-0.815847
H	-0.930513	-3.088966	2.521607
H	-1.035295	-3.228296	-1.778020
C	-0.866314	-3.044265	0.365306
H	-3.869398	-5.506674	0.518238
Cd	-0.468967	0.282415	0.251308
Br	-1.619288	2.439131	0.213948

RC E= -3.42527464

Se	-0.357436	-2.826466	-0.077682
C	-3.253883	-5.714320	-2.485789
C	-2.334793	-6.237878	-1.576899
C	-3.298611	-4.337521	-2.704325
H	-2.294691	-7.309440	-1.395880
H	-4.014859	-3.918648	-3.407302
C	-1.460564	-5.393023	-0.892031
C	-2.429238	-3.486410	-2.021287
H	-0.749577	-5.806321	-0.183037
H	-2.475491	-2.414632	-2.189322

C	-1.500662	-4.012192	-1.115739
H	-3.932965	-6.374853	-3.018701
Cd	1.521602	-2.638380	-1.690340
H	4.083133	0.029724	1.056213
O	3.338918	-0.596214	-0.563634
H	4.050138	-0.845591	-1.180072
O	3.972120	-0.875737	0.721933
Br	3.244125	-2.629212	-3.464280

TS E= -3.39559112

Nimag= -177.218

Se	-0.671391	-1.659346	-0.531625
C	-2.442283	-5.819799	-1.866242
C	-2.103517	-5.582884	-0.532941
C	-2.239641	-4.821477	-2.819288
H	-2.260396	-6.355312	0.215925
H	-2.504734	-4.995834	-3.859108
C	-1.564897	-4.357002	-0.150155
C	-1.695762	-3.593156	-2.447764
H	-1.304727	-4.178309	0.888708
H	-1.554785	-2.819800	-3.196699
C	-1.352816	-3.354491	-1.108641
H	-2.863775	-6.777346	-2.160443
Cd	1.310728	-1.153573	-2.376078
H	1.970617	-1.677967	0.240880
O	3.609672	-2.275775	-0.972579
H	3.667603	-3.242412	-0.924549
O	1.706458	-2.238844	-0.506455
Br	1.814312	-0.202387	-4.570421

PC E= -3.48173971

Se	0.301878	-3.097542	0.440347
C	-2.994039	-5.529287	-1.912639
C	-3.263959	-4.972602	-0.660840
C	-1.734543	-5.350935	-2.484916
H	-4.244554	-5.097579	-0.207425
H	-1.512950	-5.783068	-3.458299
C	-2.290159	-4.234565	0.008238
C	-0.743727	-4.633904	-1.813815
H	-2.525077	-3.782053	0.969293
H	0.242711	-4.520212	-2.252257
C	-1.021668	-4.069366	-0.565038
H	-3.759943	-6.094037	-2.437300
Cd	1.321056	-0.941447	-1.912226
H	3.721618	-2.396250	-1.374128
O	3.939344	-1.584302	-1.857529
H	4.486980	-1.862944	-2.602255
O	1.508846	-2.680271	-0.831725
Br	0.852439	1.075598	-3.188305

2-CdBr E= -2.95907744

Se	0.604002	-1.763699	0.631665
Cd	-0.052360	0.948877	-1.083321
C	-3.208736	-4.313984	-0.496157
C	-2.887029	-4.053685	0.837084
C	-2.402342	-3.797983	-1.511066
H	-3.512723	-4.444179	1.636254
H	-2.642360	-3.999434	-2.552660
C	-1.777618	-3.272669	1.155461
C	-1.273518	-3.038287	-1.204135
H	-1.556935	-3.050831	2.197227
H	-0.632296	-2.664420	-1.996303
C	-0.962165	-2.769054	0.132860
H	-4.082125	-4.912347	-0.741272
O	0.928751	-0.814359	-0.873012
Br	-1.149260	3.083472	-1.406039

TS_{iso} E= -2.94057279

Nimag= -149.905

Se	-0.619539	-1.467168	-1.229353
Br	3.575356	-1.473683	-3.854525
C	-2.516210	-5.762294	-1.912454
C	-1.366380	-5.618163	-1.135663
C	-3.100873	-4.642278	-2.507641
H	-0.914223	-6.487598	-0.664276
H	-4.001163	-4.749518	-3.107819
C	-0.788249	-4.360176	-0.956456
C	-2.537902	-3.379062	-2.329850
H	0.103259	-4.228615	-0.348893
H	-3.005165	-2.509170	-2.787150
C	-1.377854	-3.253876	-1.562823
H	-2.960369	-6.744488	-2.051112
Cd	1.424926	-1.437423	-2.706225
O	0.769485	-1.748332	-0.239216

4-CdBr E= -2.94764202

Se	0.883601	-1.864924	0.025160
Cd	-0.117166	0.523154	-0.513026
C	-3.226652	-4.220494	-0.451894
C	-2.948995	-3.553168	0.742815
C	-2.312717	-4.175117	-1.505003
H	-3.653607	-3.595840	1.569758
H	-2.523082	-4.702354	-2.432521
C	-1.761556	-2.834919	0.884094
C	-1.122002	-3.457652	-1.375481
H	-1.546473	-2.328151	1.823217
H	-0.391636	-3.423826	-2.179676
C	-0.865858	-2.785060	-0.184732
H	-4.152040	-4.780598	-0.557702
O	1.743837	-2.250846	-1.347040
Br	-0.973568	2.756166	-1.117864

5-CdBr E= -3.48434087

Se	-0.636283	-1.567061	-0.718058
Br	1.765249	-1.252963	-4.734974
C	-2.441871	-5.733400	-2.039747
C	-1.667331	-5.658864	-0.882750
C	-2.663238	-4.583043	-2.798079
H	-1.494104	-6.549670	-0.283619
H	-3.265343	-4.630093	-3.702016
C	-1.109734	-4.445024	-0.477157
C	-2.113129	-3.363686	-2.405735
H	-0.514595	-4.385166	0.428055
H	-2.292343	-2.476118	-3.008573
C	-1.334598	-3.304429	-1.246262
H	-2.872690	-6.681767	-2.349241
Cd	1.895563	-1.682674	-2.330032
H	1.251752	-2.074472	0.418236
O	2.672764	-2.023079	-0.444384
H	3.238462	-2.803998	-0.412373
O	0.304250	-1.974842	0.749723

1-HgBr E= -2.73443507

Se	0.725518	-1.981228	0.342740
Br	-1.397073	2.451714	0.034467
C	-3.037135	-4.856005	0.474246
C	-2.453192	-4.409379	1.659395
C	-2.528661	-4.428064	-0.752187
H	-2.841202	-4.741412	2.619314
H	-2.975961	-4.774985	-1.680540
C	-1.367344	-3.534074	1.622902
C	-1.440934	-3.555926	-0.797714
H	-0.914191	-3.189570	2.547373
H	-1.042871	-3.231850	-1.754383
C	-0.860967	-3.102358	0.392182
H	-3.883927	-5.536734	0.505957
Hg	-0.350597	0.234095	0.192377

RC E= -3.38992565

Se	0.621971	-1.993658	0.612536
C	-3.188120	-4.807557	0.587702
C	-2.670341	-4.332491	1.792305
C	-2.598792	-4.426605	-0.617679
H	-3.122163	-4.627102	2.736299
H	-2.994677	-4.795288	-1.560760
C	-1.568895	-3.476311	1.795829
C	-1.495572	-3.572858	-0.624189
H	-1.167242	-3.110251	2.735722
H	-1.038748	-3.286520	-1.566172
C	-0.981845	-3.092866	0.585276
H	-4.046986	-5.473651	0.588093
H	1.723979	-2.322181	-1.934471
O	3.281771	-2.969670	-2.804450
H	3.088112	-3.853574	-3.156583
O	1.953469	-2.365849	-2.881227
Hg	-0.409539	0.236820	0.335508
Br	-1.426947	2.454263	0.092328

TS E= -3.35043376

Nimag= -520.88

Se	0.701458	-1.938794	0.398635
C	-3.040249	-4.811800	0.474181
C	-2.450360	-4.386026	1.663725
C	-2.531881	-4.377809	-0.750532
H	-2.835570	-4.727547	2.620985
H	-2.983028	-4.715258	-1.680126
C	-1.355672	-3.521859	1.633233
C	-1.438476	-3.513463	-0.798863
H	-0.893243	-3.196676	2.560600
H	-1.028262	-3.194314	-1.750326
C	-0.863193	-3.087436	0.399842
H	-3.892337	-5.485929	0.501096
H	2.273522	-2.240792	-1.616309
O	2.473779	-3.096345	-3.068988
H	2.551761	-4.022298	-2.793619
O	1.358017	-2.569889	-1.470967
Hg	-0.464469	0.278281	0.159831
Br	-1.566563	2.454807	-0.096330

PC E= -3.44232339

Se	1.066072	-3.187847	0.337110
C	-3.493420	-4.494781	0.537585
C	-2.892376	-3.902217	1.649689
C	-2.763071	-4.678982	-0.636597
H	-3.456724	-3.764148	2.568478
H	-3.227930	-5.147674	-1.500545
C	-1.561899	-3.488818	1.588902
C	-1.431366	-4.267156	-0.710512
H	-1.097164	-3.034703	2.461823
H	-0.843987	-4.414757	-1.612322
C	-0.850843	-3.667529	0.402130
H	-4.529939	-4.817342	0.589552
H	2.226858	-2.311348	-2.246040
O	2.403525	-1.379201	-2.479174
H	2.052578	-1.273631	-3.371338
O	1.591795	-3.768052	-1.137152
Hg	0.671582	-0.632462	-0.049873
Br	0.198042	1.796001	-0.280649

4-HgBr E= -2.92006326

Se	0.864580	-1.850531	0.016656
Hg	-0.102987	0.526748	-0.526008
C	-3.222368	-4.229705	-0.447867
C	-2.946033	-3.555804	0.743087

C	-2.313145	-4.183788	-1.505419
H	-3.648626	-3.597939	1.571582
H	-2.525096	-4.714652	-2.430339
C	-1.762534	-2.829763	0.877372
C	-1.126084	-3.460112	-1.383019
H	-1.548294	-2.313631	1.811259
H	-0.399963	-3.424830	-2.190650
C	-0.871286	-2.783101	-0.194662
H	-4.145018	-4.795267	-0.548533
O	1.744715	-2.279420	-1.318260
Br	-0.965776	2.798168	-1.095096

TS_{iso} E= -2.89108332

Nimag= -177.558

Se	0.702080	-2.203569	0.905321
Br	0.182094	1.753963	-2.113005
C	-3.111264	-4.948925	0.428177
C	-2.890159	-4.253619	1.619256
C	-2.212470	-4.817825	-0.630759
H	-3.585066	-4.355928	2.449156
H	-2.378081	-5.362772	-1.557090
C	-1.774571	-3.429785	1.754653
C	-1.096534	-3.988397	-0.513439
H	-1.603661	-2.899981	2.689437
H	-0.386116	-3.880588	-1.327867
C	-0.888982	-3.298435	0.680344
H	-3.980832	-5.593134	0.328625
O	1.544034	-2.348586	-0.666437
Hg	0.529578	-0.236023	-0.757818

2-HgBr E= -2.91754966

Se	0.638762	-1.808262	0.579319
Hg	-0.073212	1.003800	-1.033728
C	-3.212516	-4.318198	-0.489258
C	-2.901151	-4.013379	0.836172
C	-2.388933	-3.854222	-1.516510
H	-3.542094	-4.362006	1.642429
H	-2.624489	-4.089872	-2.551770
C	-1.782150	-3.237258	1.135507
C	-1.249588	-3.105227	-1.227203
H	-1.568277	-2.974455	2.168831
H	-0.596681	-2.769154	-2.026703
C	-0.947956	-2.790766	0.102429
H	-4.093435	-4.911000	-0.720838
O	0.901152	-0.790913	-0.889074
Br	-1.166146	3.158331	-1.283017

5-HgBr E= -3.43561835

Se	-0.865122	-1.561350	-0.576538
Br	2.027544	-1.003160	-4.833984
C	-2.432072	-5.773039	-2.038460
C	-1.701082	-5.688691	-0.853573
C	-2.673528	-4.615442	-2.779889
H	-1.510447	-6.584052	-0.266121
H	-3.238850	-4.667651	-3.707364
C	-1.215061	-4.461196	-0.401853
C	-2.184681	-3.384577	-2.345609
H	-0.658053	-4.398155	0.527224
H	-2.370694	-2.494195	-2.942816
C	-1.456176	-3.308698	-1.152450
H	-2.810647	-6.731843	-2.382303
Hg	2.398852	-1.605040	-2.533377
H	1.127609	-2.052008	0.396587
O	2.769146	-2.026909	-0.586605
H	3.281773	-2.847280	-0.557724
O	0.234270	-1.987060	0.797869

6 E= -3.00552519

Se	0.523057	-1.657408	0.537644
C	-3.196423	-4.383178	-0.477188
C	-2.910569	-4.011141	0.836705
C	-2.383382	-3.923603	-1.513853
H	-3.541436	-4.355843	1.652615
H	-2.598003	-4.208025	-2.541455
C	-1.828456	-3.176721	1.113803
C	-1.282849	-3.109525	-1.247587
H	-1.635680	-2.870405	2.139652
H	-0.644986	-2.769050	-2.056802
C	-1.008425	-2.732246	0.069692
H	-4.048370	-5.022846	-0.691565
O	0.892479	-0.858077	-1.065799
H	0.348947	-0.056373	-1.082150

ZnOHCl E= -0.51211012

Zn	0.051856	0.766931	-1.037493
Cl	-0.891925	2.621632	-1.182305
O	0.894354	-0.799719	-1.045706
H	0.827122	-1.261985	-0.200974

CdOHCl E= -0.48153380

Cd	0.029573	0.805144	-1.044789
Cl	-0.994662	2.840362	-1.214068
O	0.966003	-0.948826	-1.036745
H	0.880493	-1.369822	-0.170877

HgOHCl E= -0.43824598

Hg	0.037975	0.800296	-1.057808
Cl	-0.993134	2.826089	-1.198393
O	0.975246	-0.960923	-1.047475
H	0.861320	-1.338603	-0.162802

ZnOHBr E= -0.49343561

Zn	0.067038	0.737528	-1.035946
Br	-0.938701	2.716962	-1.194139
O	0.910669	-0.833354	-1.040949
H	0.842401	-1.294277	-0.195444

CdOHBr E= -0.46575337

Cd	0.043441	0.778279	-1.043185
Br	-1.038615	2.930313	-1.225574
O	0.981157	-0.980570	-1.031874
H	0.895423	-1.401164	-0.165846

HgOHBr E= -0.42361063

Hg	0.687440	1.114297	0.000000
Br	-0.303647	3.309741	0.000000
O	1.618623	-0.660382	0.000000
H	0.929977	-1.341604	0.000000

1-ZnI E= -2.76446239

Se	1.062993	-3.065995	1.393550
Zn	0.988397	-1.200907	0.051608
C	-3.260594	-4.761214	0.408778
C	-3.075733	-3.892971	1.484276
C	-2.173506	-5.107781	-0.393052
H	-3.916471	-3.620014	2.117578
H	-2.306856	-5.787285	-1.231433
C	-1.811961	-3.368382	1.758169
C	-0.906077	-4.588977	-0.125547
H	-1.672964	-2.696191	2.599319
H	-0.061978	-4.867951	-0.748714
C	-0.724048	-3.712608	0.949275
H	-4.246376	-5.167627	0.197987
I	0.918779	0.783663	-1.378216

RC E= -3.41932275

Se	0.674707	-3.561631	-0.338491
Zn	0.061068	-1.603648	-1.389391
C	-3.568238	-4.844016	1.360988
C	-2.744009	-3.995805	2.099974
C	-3.149906	-5.292528	0.108551
H	-3.059682	-3.642955	3.078966
H	-3.784085	-5.956396	-0.474269
C	-1.508037	-3.594405	1.591766
C	-1.915859	-4.894442	-0.407248
H	-0.868332	-2.938601	2.174418
H	-1.594492	-5.249051	-1.381814
C	-1.092191	-4.039207	0.332275
H	-4.530398	-5.155122	1.759685
H	3.527039	-2.169183	-2.612697
O	3.655889	-0.379535	-2.014604
H	3.695886	0.237672	-2.763831
O	2.870468	-1.453635	-2.617075
I	-0.895508	0.421529	-2.399972

TS E= -3.39783509

Nimag= -456.697

Se	1.215628	-3.225851	0.549964
Zn	1.004414	-0.822530	-0.139677
C	-3.228902	-4.777514	0.493350
C	-2.935445	-3.561128	1.111074
C	-2.209684	-5.511726	-0.115514
H	-3.724226	-2.986440	1.589813
H	-2.432492	-6.460499	-0.597116
C	-1.630656	-3.073194	1.119799
C	-0.901382	-5.034944	-0.111331
H	-1.410363	-2.131534	1.612471
H	-0.111316	-5.607032	-0.588047
C	-0.606654	-3.809280	0.504973
H	-4.248828	-5.152882	0.487427
H	2.167423	-2.187189	-1.938582
O	1.759715	-1.187193	-3.355456
H	1.134003	-1.717124	-3.873894
O	1.250795	-2.093479	-1.611593
I	0.611290	1.462081	0.629570

PC E= -3.48398744

Se	1.076391	-2.656425	0.858867
Zn	0.477984	-0.067329	-0.655285
C	-3.108928	-4.808110	0.496912
C	-2.643096	-4.348093	1.728353
C	-2.342023	-4.605414	-0.651983
H	-3.237664	-4.492480	2.627232
H	-2.698206	-4.962717	-1.615300
C	-1.425453	-3.672757	1.811509
C	-1.106358	-3.965024	-0.576254
H	-1.088105	-3.282791	2.768650
H	-0.506071	-3.837697	-1.471152
C	-0.650340	-3.490760	0.659218
H	-4.066283	-5.318352	0.431233
H	1.741351	-2.562719	-2.555059
O	1.779786	-2.805937	-3.493100
H	2.649903	-3.208246	-3.599209
O	1.221317	-1.725781	-0.712700
I	-0.441884	2.166674	-0.624700

2-ZnI E= -2.96415035

Se	0.564417	-1.680894	0.572888
Zn	-0.010125	0.840201	-1.024788
C	-3.190871	-4.339516	-0.488762
C	-2.895134	-4.009514	0.834512
C	-2.378080	-3.858115	-1.515797
H	-3.526758	-4.371418	1.642381

H	-2.599473	-4.112013	-2.549964
C	-1.804496	-3.193416	1.131029
C	-1.267659	-3.064395	-1.230001
H	-1.604802	-2.916222	2.163599
H	-0.625149	-2.712272	-2.030810
C	-0.983085	-2.726301	0.096872
H	-4.050286	-4.964021	-0.717648
O	0.857966	-0.752047	-0.969881
I	-1.093180	2.997363	-1.187043

4-ZnI E= -2.94002874

Se	0.884470	-1.832298	0.028297
Zn	-0.108637	0.354038	-0.485219
C	-3.222412	-4.197371	-0.454967
C	-2.946512	-3.535725	0.743238
C	-2.309104	-4.143742	-1.508155
H	-3.651201	-3.584319	1.569799
H	-2.518587	-4.665901	-2.438741
C	-1.760618	-2.815522	0.888838
C	-1.120073	-3.424003	-1.374937
H	-1.546480	-2.313097	1.830407
H	-0.389833	-3.382521	-2.178945
C	-0.865772	-2.758597	-0.180051
H	-4.146365	-4.759320	-0.563642
O	1.740299	-2.190292	-1.356568
I	-1.007086	2.555041	-1.139251

1-CdI E= -2.74974885

Se	0.721884	-1.927286	0.307955
C	-3.032992	-4.824849	0.474428
C	-2.454056	-4.357759	1.654146
C	-2.519523	-4.413472	-0.755332
H	-2.843667	-4.676746	2.617971
H	-2.960479	-4.776220	-1.680804
C	-1.369949	-3.480499	1.608561
C	-1.435098	-3.537125	-0.809347
H	-0.919604	-3.125162	2.530424
H	-1.034915	-3.226855	-1.769827
C	-0.859356	-3.062265	0.374552
H	-3.877568	-5.507915	0.513111
Cd	-0.443691	0.269553	0.242494
I	-1.661004	2.599813	0.170013

RC E= -3.40547294

Se	-0.217157	-2.757254	-0.309582
C	-3.305651	-5.722655	-2.356742
C	-2.306194	-6.219437	-1.520374
C	-3.373420	-4.352862	-2.610211
H	-2.246923	-7.285347	-1.312947
H	-4.151332	-3.954615	-3.257456
C	-1.376754	-5.354754	-0.941227
C	-2.447305	-3.482277	-2.034673
H	-0.605061	-5.747371	-0.285948
H	-2.509426	-2.416193	-2.231138
C	-1.440119	-3.980785	-1.200397
H	-4.028572	-6.398492	-2.806316
Cd	1.605171	-2.731944	-2.011375
H	3.276124	-1.077998	1.123978
O	3.628813	0.144392	-0.276471
H	4.566959	0.118771	-0.528086
O	3.453101	-1.231664	0.181530
I	3.394414	-2.850231	-3.948539

TS E= -3.37818537

Nimag= -175.222

Se	-0.669929	-1.660260	-0.520620
C	-2.447351	-5.814845	-1.865787
C	-2.095229	-5.587389	-0.534287
C	-2.256372	-4.808899	-2.813179
H	-2.242955	-6.365817	0.210231
H	-2.531621	-4.975898	-3.851591
C	-1.554964	-4.363374	-0.147718
C	-1.710922	-3.582417	-2.437813
H	-1.284359	-4.191980	0.889704
H	-1.578155	-2.803217	-3.182085
C	-1.354655	-3.353177	-1.100548
H	-2.870044	-6.770963	-2.162928
Cd	1.321636	-1.159719	-2.371727
H	1.966653	-1.693191	0.248908
O	3.611150	-2.311928	-0.933804
H	3.656515	-3.278800	-0.878588
O	1.698316	-2.251152	-0.498928
I	1.862952	-0.132688	-4.726714

PC E= -3.46400577

Se	0.310616	-3.093598	0.429681
C	-2.989385	-5.535027	-1.907734
C	-3.251866	-4.986635	-0.650658
C	-1.735915	-5.345858	-2.489642
H	-4.227668	-5.120230	-0.189400
H	-1.519996	-5.771345	-3.467235
C	-2.276830	-4.246408	0.014089
C	-0.743869	-4.625987	-1.823371
H	-2.506187	-3.801091	0.979868
H	0.237812	-4.503228	-2.269891
C	-1.014444	-4.069878	-0.569278
H	-3.756285	-6.101725	-2.428835
Cd	1.338461	-0.926371	-1.925493
H	3.664586	-2.416655	-1.334428
O	3.947625	-1.627080	-1.823012
H	4.482206	-1.953511	-2.557745
O	1.512284	-2.677615	-0.846107

I	0.813106	1.247475	-3.281628

2-CdI E= -2.94149256

Se	0.575820	-1.753729	0.651813
Cd	-0.002317	0.954162	-1.106635
C	-3.207411	-4.339774	-0.493729
C	-2.883078	-4.090758	0.841223
C	-2.411748	-3.801736	-1.505428
H	-3.499698	-4.499120	1.638543
H	-2.653392	-3.994088	-2.548401
C	-1.782122	-3.300104	1.164120
C	-1.291743	-3.030381	-1.194393
H	-1.558908	-3.089824	2.207851
H	-0.658717	-2.638993	-1.984596
C	-0.978053	-2.773210	0.144136
H	-4.073950	-4.946654	-0.742160
O	0.932216	-0.838486	-0.864379
I	-1.113613	3.280112	-1.481378

4-CdI E= -2.92971487

Se	0.894178	-1.894066	0.030211
Cd	-0.099881	0.512632	-0.500792
C	-3.230298	-4.223122	-0.452902
C	-2.949806	-3.558065	0.742452
C	-2.314501	-4.183248	-1.504629
H	-3.655942	-3.596260	1.568340
H	-2.527062	-4.708668	-2.432702
C	-1.757812	-2.847875	0.885705
C	-1.119270	-3.473740	-1.373076
H	-1.540805	-2.342888	1.825355
H	-0.387655	-3.444062	-2.176304
C	-0.859786	-2.803447	-0.181593
H	-4.159253	-4.777008	-0.560273
O	1.750605	-2.278193	-1.345620
I	-1.010626	2.924380	-1.144068

1-HgI E= -2.71843903

Se	0.745481	-2.018224	0.294231
I	-1.447712	2.603123	0.137361
C	-3.043768	-4.855416	0.478997
C	-2.464664	-4.382533	1.656482
C	-2.521730	-4.464929	-0.754123
H	-2.863371	-4.685076	2.621779
H	-2.965132	-4.832192	-1.676537
C	-1.369365	-3.520077	1.605379
C	-1.426289	-3.603226	-0.814053
H	-0.918674	-3.157426	2.524101
H	-1.019554	-3.305921	-1.775768
C	-0.849476	-3.124728	0.367784
H	-3.897189	-5.527200	0.522108
Hg	-0.327094	0.218049	0.218388

RC E= -3.37458478

Se	0.811740	-2.554145	0.727674
C	-3.429848	-4.658889	0.624856
C	-2.907888	-4.180143	1.826185
C	-2.710617	-4.491072	-0.558378
H	-3.460918	-4.309058	2.753387
H	-3.109283	-4.864302	-1.498479
C	-1.672871	-3.531756	1.847720
C	-1.473117	-3.847173	-0.546849
H	-1.269373	-3.160641	2.784861
H	-0.917705	-3.726657	-1.471437
C	-0.954786	-3.362553	0.658929
H	-4.393063	-5.162290	0.610794
H	1.851102	-3.057292	-1.755257
O	2.376270	-1.873834	-3.140809
H	3.342299	-1.903505	-3.233237
O	2.123278	-3.236161	-2.675972
Hg	0.202783	-0.213568	0.137354
I	-0.441267	2.270893	-0.442277

TS E= -3.33503823

Nimag= -507.058

Se	0.721617	-1.957072	0.391804
C	-3.042084	-4.801873	0.470649
C	-2.446824	-4.382341	1.659791
C	-2.529934	-4.372806	-0.754206
H	-2.834746	-4.720170	2.617292
H	-2.985149	-4.705253	-1.683644
C	-1.343492	-3.529362	1.628632
C	-1.428272	-3.519050	-0.802968
H	-0.876993	-3.209367	2.555782
H	-1.016228	-3.202876	-1.754636
C	-0.847303	-3.098761	0.395211
H	-3.901179	-5.467034	0.498025
H	2.285541	-2.260774	-1.621104
O	2.482988	-3.139209	-3.072533
H	2.558461	-4.062334	-2.787272
O	1.372203	-2.593363	-1.476227
Hg	-0.467455	0.273109	0.183918
I	-1.685916	2.595390	-0.062445

PC E= -3.42683641

Se	1.076077	-3.212347	0.345208
C	-3.490623	-4.494944	0.527779
C	-2.889128	-3.909881	1.643640
C	-2.757775	-4.679276	-0.644866
H	-3.455334	-3.771554	2.561273
H	-3.222912	-5.142031	-1.511886
C	-1.556045	-3.504370	1.588161
C	-1.423465	-4.275251	-0.713378

H	-1.091247	-3.056128	2.464086
H	-0.834447	-4.423023	-1.614087
C	-0.841786	-3.683356	0.403089
H	-4.529219	-4.811435	0.575559
H	2.215878	-2.331764	-2.233890
O	2.387739	-1.401223	-2.479527
H	2.022400	-1.306064	-3.367068
O	1.603095	-3.786667	-1.132230
Hg	0.685312	-0.631681	-0.035908
I	0.165215	1.976850	-0.252886

4-HgI E= -2.90450901

Se	0.882815	-1.887644	0.014305
Hg	-0.088702	0.517915	-0.507051
C	-3.226699	-4.227543	-0.449383
C	-2.948065	-3.548063	0.737964
C	-2.312206	-4.199096	-1.502954
H	-3.655080	-3.575822	1.563327
H	-2.525854	-4.734216	-2.425057
C	-1.757169	-2.834505	0.872483
C	-1.117875	-3.487458	-1.380394
H	-1.541832	-2.313727	1.803550
H	-0.388355	-3.464723	-2.185409
C	-0.859990	-2.805190	-0.195523
H	-4.155214	-4.783398	-0.550241
O	1.753254	-2.312166	-1.329392
I	-1.026943	2.962008	-1.086123

2-HgI E= -2.90245251

Se	0.658889	-1.842551	0.601267
Hg	-0.054378	0.962651	-1.047912
C	-3.219481	-4.300632	-0.493231
C	-2.902004	-4.018174	0.835884
C	-2.391417	-3.833062	-1.515087
H	-3.546236	-4.369624	1.638312
H	-2.631190	-4.051411	-2.553197
C	-1.772662	-3.261043	1.144108
C	-1.242715	-3.101731	-1.217269
H	-1.554045	-3.015599	2.180771
H	-0.586907	-2.762136	-2.012883
C	-0.934893	-2.809548	0.115970
H	-4.108382	-4.878663	-0.731512
O	0.942603	-0.835288	-0.864700
I	-1.263895	3.254232	-1.353937

Table S7 Coordinates (Å) and energies (E, Hartree) of stationary points and number of imaginary frequencies (Nimag, cm^{-1}) of transition states. Level of theory: COSMO-ZORA-OLYP/TZ2P.

H_2O_2 E= -0.66271837

O	-0.038924	-0.728562	1.729852
O	0.038924	0.728562	1.729852
H	0.774511	0.875800	2.349402
H	-0.774511	-0.875800	2.349402

H_2O E= -0.52411819

O	0.000000	0.000000	-0.208137
H	0.000000	0.759715	0.390751
H	0.000000	-0.759715	0.390751

1-ZnCl E= -2.81512517

Se	0.763391	-1.336252	-0.451921
Zn	-0.367689	0.664006	-0.457177
Cl	-1.428502	2.530016	-0.458718
C	-2.893734	-4.364123	-0.569128
C	-2.526799	-3.760578	0.634949
C	-2.202389	-4.046127	-1.738971
H	-3.057254	-4.003372	1.552590
H	-2.478816	-4.511561	-2.682023
C	-1.473065	-2.845066	0.673471
C	-1.150592	-3.127504	-1.709477
H	-1.189801	-2.387416	1.616550
H	-0.619493	-2.884873	-2.625030
C	-0.784694	-2.522922	-0.501706
H	-3.712036	-5.079048	-0.594951

RC E= -3.47664689

Se	0.838515	-3.347346	-0.156252
Zn	0.258660	-1.478031	-1.402941
Cl	-1.067855	0.024100	-2.260233
C	-3.381811	-5.016340	1.238761
C	-3.085922	-3.664358	1.422954
C	-2.442318	-5.846687	0.626340
H	-3.807701	-3.007139	1.902251
H	-2.658419	-6.902074	0.477841
C	-1.859978	-3.143993	1.004370
C	-1.219980	-5.330266	0.190960
H	-1.638779	-2.094873	1.176005
H	-0.502090	-5.985157	-0.294570
C	-0.922632	-3.974074	0.376808
H	-4.335289	-5.418621	1.570869
H	2.083123	-0.007713	-2.955071
O	3.212221	-1.519489	-2.669954
H	4.024247	-1.180155	-2.252405
O	2.239368	-0.560401	-2.168050

TS E= -3.46111923

Nimag= -187.864

Se	1.256404	-3.325941	0.538968
Zn	1.138580	-0.958466	0.108759
Cl	0.885197	1.118483	0.598803
C	-3.221331	-4.772144	0.448752
C	-2.915427	-3.542949	1.037384
C	-2.206293	-5.542988	-0.122473
H	-3.700613	-2.941661	1.487989
H	-2.438524	-6.501216	-0.579723
C	-1.601675	-3.079288	1.055871
C	-0.888853	-5.089592	-0.110943
H	-1.373852	-2.130727	1.531273
H	-0.103753	-5.692275	-0.557428
C	-0.583196	-3.851577	0.476806
H	-4.247630	-5.129050	0.437489
H	2.246020	-2.272020	-1.757548
O	1.496429	-1.060871	-3.316391
H	0.874582	-1.671164	-3.742138
O	1.318793	-2.020171	-1.614768

PC E= -3.54851867

Se	0.670953	-2.548508	1.152841
Zn	1.226507	-0.944327	-1.489565
Cl	0.493528	1.069979	-1.788101
C	-3.326513	-5.017820	0.611071
C	-2.937411	-4.525892	1.861158
C	-2.520038	-4.772071	-0.501007
H	-3.555354	-4.709445	2.737155
H	-2.810199	-5.150120	-1.478945
C	-1.758178	-3.796201	1.998821
C	-1.334027	-4.043947	-0.375835
H	-1.472550	-3.420139	2.979073
H	-0.711289	-3.864160	-1.246182
C	-0.951250	-3.552417	0.876418
H	-4.247120	-5.586048	0.507826
H	2.590496	-2.378934	-3.321299
O	2.494753	-1.431253	-3.143395
H	2.244024	-1.037106	-3.992220
O	1.369489	-2.518054	-0.511908

2-ZnCl E= -3.01856670

Se	0.195918	-1.406752	0.786475
Zn	0.304743	0.867867	-1.291713
Cl	-0.236816	2.809561	-1.927371
C	-3.144258	-4.498332	-0.492719
C	-2.812482	-4.295949	0.850450
C	-2.478263	-3.769799	-1.478924
H	-3.321416	-4.858068	1.630027
H	-2.725352	-3.918860	-2.527704
C	-1.827680	-3.375714	1.205300
C	-1.487876	-2.845269	-1.137124
H	-1.581790	-3.232747	2.255409
H	-0.972047	-2.290317	-1.913691
C	-1.162786	-2.646232	0.207979
H	-3.911884	-5.217690	-0.765294
O	0.827340	-0.805952	-0.808892

TS_{iso} E= -2.99647271

Nimag= -175.825

Se	0.531328	-1.449385	0.366208
Zn	-0.135394	0.614361	-0.475779
Cl	-0.581600	2.434829	-1.499792
C	-3.154210	-4.311289	-0.470720
C	-2.877923	-3.873439	0.827140
C	-2.356788	-3.885610	-1.534486
H	-3.492497	-4.206748	1.659611
H	-2.565803	-4.227703	-2.545190
C	-1.805062	-3.013598	1.065773
C	-1.285941	-3.016880	-1.310191
H	-1.586710	-2.686645	2.079570
H	-0.660821	-2.681328	-2.132453
C	-1.024991	-2.587538	-0.011413
H	-3.986849	-4.986158	-0.650701
O	1.309195	-1.230426	-1.187911

4-ZnCl E= -3.00482193

Se	0.864920	-1.845200	0.038875
Zn	-0.052544	0.337619	-0.447105
Cl	-0.811604	2.287130	-0.958239
C	-3.224641	-4.223723	-0.449776
C	-2.946617	-3.543272	0.738519
C	-2.313851	-4.181628	-1.506603
H	-3.649806	-3.578713	1.566815
H	-2.524159	-4.714032	-2.431039
C	-1.760213	-2.820587	0.873737
C	-1.125237	-3.457150	-1.384494
H	-1.545525	-2.302040	1.805291
H	-0.408154	-3.424309	-2.199611
C	-0.866041	-2.779011	-0.197311
H	-4.147513	-4.789160	-0.548710
O	1.755320	-2.234257	-1.346471

1-CdCl E= -2.79845908

Se	0.728902	-1.970588	0.337619
Cl	-1.379265	2.386727	0.053099
C	-3.037410	-4.858265	0.473596
C	-2.455768	-4.404021	1.658325
C	-2.526941	-4.430772	-0.753357
H	-2.844048	-4.732325	2.619538
H	-2.970936	-4.780533	-1.682348
C	-1.370536	-3.525870	1.620134
C	-1.440775	-3.554390	-0.799131

H	-0.924077	-3.178845	2.547238
H	-1.047969	-3.231592	-1.758707
C	-0.860275	-3.095375	0.389501
H	-3.881735	-5.542022	0.506063
Cd	-0.357706	0.262091	0.194558

RC E= -3.45973608

Se	-0.329260	-2.104689	-1.559244
Cl	3.306114	-4.752104	0.262551
C	-3.359120	-5.697981	-2.235177
C	-3.508698	-4.840226	-1.144512
C	-2.290980	-5.513506	-3.115251
H	-4.337548	-4.973201	-0.453250
H	-2.166361	-6.174065	-3.970034
C	-2.594655	-3.806282	-0.929062
C	-1.377576	-4.477729	-2.910022
H	-2.717622	-3.147057	-0.074833
H	-0.556143	-4.338179	-3.606540
C	-1.522849	-3.621888	-1.811032
H	-4.070119	-6.503560	-2.399311
Cd	1.653874	-3.297623	-0.622054
H	3.865113	-0.936923	-1.589363
O	4.283330	-0.806637	0.261919
H	4.256397	0.149113	0.444365
O	3.292721	-0.908046	-0.802480

TS E= -3.43986731

Nimag= -232.797

Se	-0.643502	-1.701294	-0.520934
Cl	1.890478	0.075555	-4.319415
C	-2.362842	-5.868916	-1.893699
C	-2.042394	-5.622750	-0.557132
C	-2.153625	-4.875265	-2.851917
H	-2.203921	-6.390574	0.195057
H	-2.404698	-5.057561	-3.893557
C	-1.512448	-4.391133	-0.175724
C	-1.624472	-3.639511	-2.481957
H	-1.265424	-4.208064	0.865793
H	-1.483236	-2.871768	-3.235995
C	-1.298206	-3.395527	-1.140947
H	-2.775771	-6.830236	-2.187226
Cd	0.809319	-0.856173	-2.447301
H	1.930593	-1.652839	0.097841
O	3.633840	-2.602087	-0.671803
H	3.473791	-3.504504	-0.356861
O	1.736340	-2.217537	-0.667949

PC E= -3.51975675

Se	-0.099171	-2.226726	0.042575
Cl	0.409488	1.296234	-4.133714
C	-2.608549	-5.754895	-1.858006
C	-3.009476	-5.134011	-0.671123
C	-1.448585	-5.319961	-2.500517
H	-3.913115	-5.460407	-0.161613
H	-1.125515	-5.795651	-3.423845
C	-2.264312	-4.085156	-0.134826
C	-0.685497	-4.278804	-1.966149
H	-2.598426	-3.603853	0.781807
H	0.222606	-3.958267	-2.466381
C	-1.095326	-3.657595	-0.782241
H	-3.195983	-6.567884	-2.276337
Cd	0.767415	-0.387276	-2.570906
H	2.958640	-2.455231	-0.894522
O	3.898631	-2.651697	-0.713547
H	4.096245	-3.411579	-1.277321
O	1.149153	-1.876627	-1.217860

2-CdCl E= -2.99357963

Se	-0.936585	-1.325668	-1.503243
Cl	4.146893	-1.576892	-3.581324
C	-2.516424	-5.766473	-1.898613
C	-1.254799	-5.537413	-1.348089
C	-3.284938	-4.679579	-2.327336
H	-0.647739	-6.373969	-1.008949
H	-4.269576	-4.843068	-2.759268
C	-0.758637	-4.237636	-1.218377
C	-2.796846	-3.379134	-2.213359
H	0.217829	-4.070815	-0.775097
H	-3.404298	-2.546258	-2.561277
C	-1.529157	-3.154948	-1.654598
H	-2.898843	-6.779319	-1.993227
Cd	2.299412	-1.551613	-2.165352
O	0.732949	-1.529187	-0.851164

TS_{iso} E= -2.97918309

Nimag= -134.961

Se	0.427698	-1.440810	0.530901
Cd	-0.083818	0.783918	-0.573672
Cl	-0.489347	2.799916	-1.708369
C	-3.129007	-4.382387	-0.528394
C	-2.919909	-3.963446	0.788369
C	-2.295467	-3.916138	-1.546670
H	-3.561826	-4.327521	1.586736
H	-2.451346	-4.242298	-2.572226
C	-1.880437	-3.083333	1.090304
C	-1.257796	-3.026480	-1.258955
H	-1.715948	-2.772153	2.119229
H	-0.607311	-2.659915	-2.047540
C	-1.061449	-2.614953	0.058436
H	-3.936663	-5.072382	-0.758056
O	1.288561	-1.189575	-1.000427

4-CdCl E= -2.99171554

Se	-0.650507	-1.547284	-0.775097
Cl	2.927684	-1.360843	-4.254343
C	-2.487963	-5.759079	-1.992440
C	-1.552170	-5.682459	-0.959451
C	-2.861079	-4.602990	-2.682893
H	-1.263174	-6.578986	-0.416317
H	-3.595203	-4.656281	-3.482913
C	-0.981081	-4.454737	-0.615208
C	-2.300241	-3.370665	-2.344610
H	-0.256916	-4.385998	0.191397
H	-2.602770	-2.473482	-2.879899
C	-1.356288	-3.312882	-1.317441
H	-2.930077	-6.716136	-2.256569
Cd	1.195439	-1.458001	-2.627203
O	0.187948	-1.850028	0.659945

1-HgCl E= -2.75779624

Se	0.707287	-1.956089	0.353982
Cl	-1.347729	2.390197	0.014093
C	-3.031035	-4.865374	0.473196
C	-2.450055	-4.418576	1.661018
C	-2.529309	-4.424170	-0.752660
H	-2.833112	-4.759255	2.619794
H	-2.974544	-4.769786	-1.682391
C	-1.372938	-3.530722	1.627649
C	-1.450239	-3.539602	-0.794894
H	-0.926014	-3.186446	2.555337
H	-1.061636	-3.205204	-1.751996
C	-0.872965	-3.087934	0.397668
H	-3.870183	-5.555581	0.502338
Hg	-0.356066	0.252767	0.182996

RC E= -3.42152761

Se	0.857419	-2.506697	0.564289
Cl	-0.389955	2.077564	-0.239335
C	-3.333360	-4.715341	0.495683
C	-2.785870	-4.276472	1.702091
C	-2.657819	-4.472353	-0.701524
H	-3.304885	-4.462877	2.638976
H	-3.076881	-4.812660	-1.645158
C	-1.567759	-3.594397	1.715651
C	-1.438277	-3.793280	-0.697212
H	-1.147483	-3.255256	2.657541
H	-0.919516	-3.610958	-1.632862
C	-0.894058	-3.350903	0.513505
H	-4.282236	-5.245293	0.488280
H	2.105286	-3.135389	-1.682475
O	2.115902	-2.788162	-3.562766
H	2.770543	-2.077831	-3.678081
O	2.663642	-3.488805	-2.406269
Hg	0.209089	-0.170023	0.148096

TS E= -3.39862870

Nimag= -294.828

Se	0.432012	-1.628979	0.439159
Cl	-1.895780	2.523713	-0.307310
C	-2.997431	-4.865032	0.459391
C	-2.079061	-4.774403	1.505173
C	-2.923025	-3.971873	-0.612023
H	-2.128989	-5.466343	2.341769
H	-3.634863	-4.039307	-1.430588
C	-1.089334	-3.789920	1.488724
C	-1.934945	-2.988580	-0.644917
H	-0.380763	-3.719592	2.308474
H	-1.878103	-2.309147	-1.488748
C	-1.023773	-2.899041	0.412062
H	-3.769813	-5.629194	0.477510
H	2.027220	-1.696898	-1.525913
O	1.944578	-2.777411	-3.273682
H	1.986356	-3.702285	-2.989127
O	1.217409	-2.217394	-1.654545
Hg	-0.786833	0.490781	0.047912

PC E= -3.48155796

Se	0.925414	-2.452793	0.675557
Cl	-0.748280	1.968583	-0.849658
C	-3.147444	-4.869553	0.481760
C	-2.861066	-4.056355	1.580737
C	-2.246455	-4.953642	-0.581375
H	-3.557255	-3.994954	2.413018
H	-2.464912	-5.590942	-1.434429
C	-1.674783	-3.322690	1.620065
C	-1.057763	-4.221494	-0.556321
H	-1.451911	-2.697544	2.481287
H	-0.349167	-4.288075	-1.375694
C	-0.792466	-3.409517	0.542293
H	-4.071144	-5.441460	0.456628
H	1.834836	-2.637277	-2.466228
O	1.838779	-2.504875	-3.435226
H	2.584619	-1.909654	-3.589068
O	1.833069	-3.040243	-0.607537
Hg	0.026347	-0.173063	-0.117550

4-HgCl E= -2.95532633

Se	1.032656	-2.633827	0.652724
Cl	-0.358598	1.714588	-1.349211
C	-3.160064	-4.844872	0.459277
C	-2.866275	-3.984461	1.519706
C	-2.230605	-5.037474	-0.564848
H	-3.585052	-3.838526	2.321814
H	-2.455923	-5.710708	-1.388099

C	-1.643992	-3.311885	1.559157
C	-1.005793	-4.367459	-0.538698
H	-1.416071	-2.649173	2.390522
H	-0.274653	-4.516248	-1.327699
C	-0.732240	-3.507518	0.520950
H	-4.111997	-5.368586	0.433821
O	1.950330	-3.371785	-0.534472
Hg	0.277394	-0.388460	-0.377151

TS_{iso} E= -2.91875148

Nimag= -188.092

Se	0.629432	-2.126495	0.964742
Cl	0.221207	1.645176	-2.141115
C	-3.101056	-4.967382	0.420552
C	-2.882986	-4.292516	1.624983
C	-2.215851	-4.790505	-0.644434
H	-3.565635	-4.429028	2.459966
H	-2.378864	-5.315164	-1.582781
C	-1.783668	-3.447242	1.769388
C	-1.117815	-3.937212	-0.518348
H	-1.614081	-2.936336	2.714153
H	-0.429963	-3.795613	-1.345998
C	-0.909459	-3.270700	0.690129
H	-3.956149	-5.629676	0.314739
O	1.565469	-2.315912	-0.583873
Hg	0.589468	-0.255000	-0.853549

2-HgCl E= -2.94249720

Se	0.792211	-2.338271	1.140961
Cl	-0.216307	1.749220	-2.375674
C	-3.134081	-4.870624	0.427560
C	-2.813445	-4.370178	1.692324
C	-2.288621	-4.605844	-0.651300
H	-3.467628	-4.564061	2.539031
H	-2.528497	-4.991036	-1.639721
C	-1.664226	-3.602209	1.877433
C	-1.122703	-3.858121	-0.474150
H	-1.440455	-3.198761	2.862324
H	-0.462018	-3.675185	-1.315473
C	-0.812129	-3.351523	0.792194
H	-4.036492	-5.459148	0.284767
O	1.312977	-1.883654	-0.530083
Hg	0.568745	-0.174396	-1.363307

7-ZnCl E= -3.48340971

Se	0.754528	-1.394943	-0.139056
Zn	-0.059754	0.563486	-1.221372
Cl	-0.800093	2.308624	-2.159922
C	-2.981272	-4.275065	-0.320510
C	-2.389399	-3.924140	0.892037
C	-2.471729	-3.777525	-1.522426
H	-2.779072	-4.313714	1.828337
H	-2.931781	-4.051534	-2.467795
C	-1.280511	-3.075131	0.912462
C	-1.371308	-2.921364	-1.521029
H	-0.813498	-2.809472	1.855889
H	-0.975830	-2.542843	-2.457716
C	-0.793392	-2.583517	-0.298773
H	-3.840266	-4.940253	-0.331659
S	2.129246	-1.986224	-1.781278
C	3.182808	-3.184209	-0.916422
H	2.615890	-4.065565	-0.615654
H	3.917355	-3.467553	-1.676600
H	3.693672	-2.722341	-0.071490

MeS^{-} E= -0.97168186

S	0.000000	0.000000	0.105023
C	0.000000	0.000000	-1.738526
H	1.022206	0.000000	-2.132957
H	-0.511103	-0.885256	-2.132957
H	-0.511103	0.885256	-2.132957

MeSSMe E= -1.70401259

S	-1.426497	1.523303	-0.057551
C	-0.682435	3.065661	-0.680752
H	-1.367800	3.594806	-1.344989
H	-0.371654	3.705376	0.147074
H	0.197845	2.746378	-1.247430
S	-2.993149	2.106183	1.120373
C	-4.413314	2.286229	-0.007331
H	-4.612760	1.348316	-0.528521
H	-5.260073	2.532307	0.641092
H	-4.253424	3.099289	-0.717458

8 E= -3.50998054

Se	0.817726	-1.685937	0.419360
C	-2.991311	-4.311777	-0.634012
C	-2.727667	-3.884026	0.668834
C	-2.105151	-3.976727	-1.659317
H	-3.406940	-4.141293	1.478082
H	-2.299772	-4.304450	-2.677958
C	-1.585480	-3.131727	0.947536
C	-0.964849	-3.215795	-1.392716
H	-1.387632	-2.815721	1.969176
H	-0.285243	-2.956207	-2.198568
C	-0.705038	-2.794902	-0.087986
H	-3.877912	-4.903005	-0.847412
S	2.179500	-1.768816	-1.293165
C	3.195811	-3.257963	-1.026436
H	3.723709	-3.203185	-0.073323
H	2.589352	-4.163006	-1.084252
H	3.920713	-3.252886	-1.846734

MeSZnCl E= -1.00734417

Zn	-0.087781	-0.021137	-0.409642
Cl	-0.197658	0.516559	-2.477565
S	0.068362	-0.586150	1.680481
C	-1.545844	-0.049251	2.379450
H	-2.376685	-0.570484	1.902518
H	-1.513693	-0.319349	3.437318
H	-1.676378	1.029814	2.289672

7-ZnBr E= -3.46396406

Se	0.759566	-1.399026	-0.127111
Zn	-0.057654	0.569196	-1.214872
Br	-0.847830	2.414756	-2.232267
C	-2.977177	-4.278134	-0.321051
C	-2.385236	-3.933063	0.893173
C	-2.466997	-3.775293	-1.520470
H	-2.775308	-4.326781	1.827591
H	-2.927085	-4.044450	-2.467235
C	-1.275881	-3.084747	0.917671
C	-1.366012	-2.919874	-1.514681
H	-0.808992	-2.823798	1.862491
H	-0.970155	-2.537269	-2.449549
C	-0.787636	-2.587675	-0.290988
H	-3.836616	-4.942697	-0.335438
S	2.134115	-1.988409	-1.769675
C	3.180128	-3.199006	-0.913019
H	2.607692	-4.079074	-0.618952
H	3.913747	-3.481402	-1.674443
H	3.692926	-2.746537	-0.064149

1-ZnBr E= -2.79494490

Se	0.770867	-1.700007	-0.110436
Zn	-0.041930	0.378914	-0.683169
C	-3.227044	-4.245221	-0.425029
C	-2.886749	-3.618883	0.775016
C	-2.402505	-4.092783	-1.540860
H	-3.520789	-3.733098	1.650997
H	-2.657426	-4.577945	-2.480037
C	-1.728929	-2.842848	0.862508
C	-1.243184	-3.318065	-1.461212
H	-1.470488	-2.362199	1.801293
H	-0.606752	-3.207164	-2.334022
C	-0.905236	-2.687844	-0.258232
H	-4.128089	-4.849663	-0.489724
Br	-0.859274	2.435019	-1.250457

MeSZnBr E= -0.98699805

Zn	-0.077625	-0.031482	-0.389651
Br	-0.186243	0.540607	-2.590722
S	0.066918	-0.593555	1.708605
C	-1.550005	-0.051798	2.397278
H	-2.379121	-0.573871	1.918117
H	-1.524613	-0.316936	3.456628
H	-1.678990	1.027038	2.301975

$PhSe^{-}$ E= -2.78899753

C	12.551618	8.838723	0.000000
C	11.603991	7.814036	0.000000
C	13.918450	8.546014	0.000000
H	10.546512	8.067197	0.000000
H	14.655868	9.344804	0.000000
C	11.990384	6.457513	0.000000
C	14.319313	7.206922	0.000000
H	15.378506	6.955600	0.000000
C	13.373419	6.180637	0.000000
H	13.710231	5.146753	0.000000
H	12.216671	9.874516	0.000000
Se	10.686466	5.045172	0.000000
